# Functional Foods and Micro- and Nanoplastics: Advances in Precision Nutritional Medicine for Oral–Gut–Brain Axis Health

**DOI:** 10.3390/antiox15080951

**Published:** 2026-07-30

**Authors:** Scuto Maria Concetta, Lombardo Cinzia, Zerbo Giulia, Ledda Caterina, Isola Gaetano, Musso Nicolò, Trovato Salinaro Angela

**Affiliations:** 1 Department of Medicine and Surgery, Kore University of Enna, 94100 Enna, Italy; mariaconcetta.scuto@unikore.it (S.M.C.);; 2 Department of Biomedical and Biotechnological Sciences, School of Medicine, University of Catania, 95123 Catania, Italy; cinzia.lombardo@unict.it (L.C.); giulia.zerbo@unict.it (Z.G.); 3 Department of Clinical and Experimental Medicine, University of Catania, 95123 Catania, Italy; 4 Department of General Surgery and Medical-Surgical Specialities, Unit of Periodontology, University of Catania, 95123 Catania, Italy; gaetano.isola@unict.it

**Keywords:** micro- and nanoplastics, polyphenols, probiotics, autophagy, oral–gut–brain axis, periodontitis, inflammatory bowel diseases, Alzheimer’s disease, precision nutritional medicine

## Abstract

Microplastics and nanoplastics (MNPs) are emerging environmental pollutants due to their persistence and bodily accumulation. Recently, functional foods have received much attention for their ability to reverse or block MNP damage for therapeutic purposes and the potential risk of developing oral–gut–brain axis disorders. Among these, artichoke, spirulina algae, *Opuntia ficus-indica*, pterostilbene, hydroxycinnamic acids, and quinic acid are rich sources of polyphenols. These bioactive ingredients, especially when combined with probiotics and prebiotics, exhibit significant antioxidant and anti-inflammatory potential by activating nuclear factor erythroid 2-related factor 2 (Nrf2) signaling and cellular resilience enzymes. Nrf2 activation enhances cellular resilience response, and it may preserve oral epithelial barrier (OEB), intestinal epithelial barrier (IEB), and blood–brain barrier (BBB) integrity, while modulating oral pathogens, gut microbial dysbiosis, and neuroinflammatory processes. However, most of the available evidence supporting these mechanisms derives from in vitro and animal studies, whereas clinical evidence in humans remains limited. Perturbations of Nrf2 due to circulating MNPs may exacerbate selective susceptibility to oral, gut, and nervous system disorders, including Alzheimer’s disease (AD). Although these findings are biologically plausible, the causal relationships and their clinical relevance have not yet been fully established. This review discusses the role of functional foods in maintaining oral–gut–brain health through Nrf2-mediated mechanisms that may mitigate MNP-induced inflammation and reactive oxygen species (ROS). The review also examines emerging concepts in precision nutritional medicine, including individual variability in dietary responses, microbiome-related factors, and future personalized strategies for populations exposed to MNPs. Finally, current knowledge gaps, the scarcity of human studies, and the challenges in translating preclinical findings into clinical practice are highlighted, emphasizing the need for further translational and clinical research.

## 1. Introduction

Over the 20th century the large expansion of plastic manufacturing has dramatically increased emerging environmental pollutants, particularly microplastics and nanoplastics (MNPs), which currently represent a major concern for public health in virtue of their persistence and bioaccumulation [1]. Microplastics (MPs) usually refer to tiny plastic particles from 0.1 μm to 5 mm in diameter, while nanoplastics (NPs) represent submicron-sized particles that are <0.1 μm. These NMPs, particularly, polypropylene (PP), polyethylene (PE), polyvinyl chloride (PVC), polyethylene terephthalate (PET), and polystyrene (PS), are non-biodegradable and have the ability to accumulate in higher levels in both marine and terrestrial ecosystems, including oceans, rivers, air, food, drinking water, and sediments [2]. The National Oceanic and Atmospheric Administration defines as MNPs all microbeads, capsules, fibers, or pellets of a particle size range from 1 nm to less than 5 mm. After oral ingestion and intestinal absorption, MNPs can enter the body, pass into the blood, and cross impermeable barriers such as the oral epithelial barrier (OEB), intestinal epithelial barrier (IEB), and blood–brain barrier (BBB) to reach the brain. Alterations in oral and intestinal microbiota due to MNP exposure may negatively impact the oral–gut–brain axis and contribute to the etiology and progression of nervous system disorders, particularly AD [3,4]. MNPs enter cells primarily by macropinocytosis and clathrin-mediated endocytosis in which the cell membrane engulfs the particle and brings it into the cell without the formation of a phagosome [5]. On the other hand, particles less than 0.5 μm in diameter can potentially cross lipid bilayers through vesicular transport known as transcytosis [6].

### 1.1. Micro- and Nanoplastic-Induced Damage to the Oral–Gut–Brain Axis

BBB plays an important role in the defense of the brain against potentially environmental toxins [7]. The main components of the BBB are brain microvascular endothelial cells (BMECs), pericytes, and astrocytes. BMECs are interconnected by tight junction proteins (TJs), which form a continuous sheet lining the inner surface of the capillaries. Reducing TJ protein expression directly causes increased BBB permeability, which is an important indicator of BBB impairment [7]. Emerging evidence confirms that, in mammals, ultrafine plastics cross the BBB, inducing damage and neurotoxicity and leading to cognitive impairment, likely through microglial activation in a dose-dependent manner [8]. Similarly, plastic particles promote the activation of inflammatory cytokine cascade such as IL-8, IL-6, IL-1 beta, and TNF-α and genes related to phase II of xenobiotic metabolism NAT2, SOD2, CAT, SULT1A1, and UGT2B4, upregulated in both intestinal and liver cells [9]. Oral health influences the stomach by affecting masticatory and digestive functions, and ultimately the brain function. Insufficient toothbrushing is related to increased oral dysbiosis risk, which may be attributed to the accumulation of circulating MPs and carcinogenic metabolites [10]. Deficient mastication modifies the host’s nutrient bio-accessibility and results in higher contents of harmful non-digested nitrogen at the end of gastrointestinal digestion, which is possibly fermented by microbes. Considering the importance of oral, gut, and brain health and the fact that the damage induced by MNPs to barriers could be irreversible, careful reflection and investigation on the potential harmful impact of MNPs on human health are required. Therefore, there is an urgent need to research and identify substances that interfere with MNPs to protect barrier integrity from external insults and preserve the oral–gut–brain axis from environmental pollutants, which is a significant challenge for modern civilization. Nowadays, nutritional approaches with active food nutrients are considered innovative, preventive, and therapeutic interventions to explore the cellular and molecular mechanisms of action of MNPs and to inhibit their adverse effects such as barrier dysfunction, neurotoxicity, inflammation, and gut–brain axis damage and disorders [11]. Recent evidence shows that MNPs contribute to increased risk of oral, gut, and brain damage and the onset of related disorders in vitro, in vivo, and in humans. In line with this, a recent study demonstrated that exposure of 100 μg/L of carboxyl-modified polystyrene microplastics (PS-COOH) induced neurotoxicity by affecting the neurotransmission of dopamine, glutamate, serotonin, and GABA in *C. elegans* [12]. Similarly, recent evidence suggested that plastic pollutants are positively associated with periodontitis and worsen inflammation by activating the NF-κB pathway [13]. In addition, MP exposure may be related to the potential risk of inflammatory bowel disease (IBD) in humans. Indeed, a study observed a higher concentration of fecal MPs correlated with the severity of IBD in patients compared to healthy individuals [14].

### 1.2. Nrf2 Activation as a Key Mechanism of Cellular Resilience

Recent evidence highlighted the pivotal role of functional foods in activating Nrf2 pathways and stress resilience genes to mitigate oxidative stress and promote overall health [15]. The redox-sensitive transcription factor Nrf2, in response to various exogenous insults, regulates the expression of antioxidant-responsive element (ARE)-containing resilience genes in their promoters [16,17]. In normal cellular conditions, Nrf2 is highly controlled by the repressor protein Keap1 (Kelch-like, ECH-associated protein 1), in the cytoplasm, which promotes its degradation. Under stressors, ROS or pollutants modify Keap1, leading to the dissociation of Nrf2 which translocates into the nucleus where it binds to AREs in the DNA and initiates the transcription of phase II resilience genes involved in detoxification, antioxidant defense, and regulation of inflammation. These resilience genes encode for heme oxygenase-1 (HO-1), heat shock protein 70 (Hsp70), sirtuin-1 (Sirt1), the thioredoxin (Trx)/thioredoxin reductase system, NADPH quinone oxidoreductase 1 (NQO1), γ-glutamylcysteine synthetase (γ-GCs), superoxide dismutase (SOD), catalase (CAT), glutathione (GSH), glutathione peroxidase (GPx), and glutathione reductase (GSR) to preserve cellular homeostasis and protect against the onset and progression of oral, intestinal, and neurodegenerative disorders [15]. Therefore, Nrf2 activation is essential for cellular resilience and stress adaptation against chronic oxidative damage, ultimately supporting longevity and overall health. Several studies suggested that functional foods targeting the Nrf2 pathway and stress resilience genes and their intricate interactions with other critical signaling pathways, including AMPK, NF-κB, and PI3K/Akt, can potentially prevent and treat oral–gut–brain axis disorders.

### 1.3. Nutritional Approaches to Mitigate MNP-Induced Toxicity

Synergistic supplementation with melatonin and probiotics may have application value in the prevention of neurotoxicity as it significantly reduces intestinal injury, restoring the expression of hippocampal circadian rhythm-related genes (i.e., Camk2g, Adcyap1, and Per1) and neuroplasticity molecules (i.e., 5-HT, AChE, GABA, BDNF, and CREB) that positively affect the learning and memory ability of mice [18]. Of note, long-term exposure to amino-modified polystyrene MNPs (40 mg/Kg/day) induced neurotoxic effects by upregulating AD-associated genes such as APP and MAPT and BBB damage via the activation of the toll-like receptor 2 (TLR2)/matrix metalloproteinase 9 (MMP9) signaling pathway in mice. Interestingly, the functional food camellia pollen treatment at a moderate dose of 50 mg significantly mitigated neurotoxicity and neuronal apoptosis by targeting the p53/Bax/Bcl-2 axis in mice after 15 weeks [19]. Also, in neuronal cells, camellia pollen treatment at a dose of 40 μg/mL reversed the reduction in cell viability caused by amino-modified polystyrene NPs and increased the expression of occludin and ZO-1 through inhibition of the TLR2/MMP9 axis. Moreover, the same study has shown that the camellia pollen treatment attenuated NP-induced neuronal damage by reducing the GAPDH/Ac-Tau pathway and increasing the Sirt-1 pathway in hippocampal neuron cells [19]. Other recent studies reported that *Camellia sinensis* L. bee pollen extract and its active phenolic compounds such as gallic acid and kaempferol at a dose of 100 μg/mL exerted antagonist effects, interacting with the main glucose transporters SGLT1 and GLUT2 to inhibit the activity of glucose uptake and transport in human intestinal cells [20]. This review aims to explore the antioxidant and anti-inflammatory effects of functional foods, also in synergy with probiotics, in mitigating and/or detoxifying MNP-induced damage to cells, tissues, and specific biological barriers to ultimately enhance the oral–gut–brain axis health. Furthermore, we elucidate the intricate interplay of the oral–gut–brain axis, highlighting the molecular mechanisms and therapeutic targets modulated by functional nutrients, particularly the Nrf2 pathway and stress resilience genes based on innovative platforms. Limited clinical trial evidence suggests growing interest in the use of these compounds in inflammatory bowel and neurodegenerative diseases. Finally, this review provides a basis for developing effective interventions to reduce MNP-related health risks and guide future clinical nutritional studies for the promotion of precision medicine, as well as for the adoption of protective public health measures.

## 2. Narrative Review Strategy and the Selection Criteria for Functional Nutrients

A comprehensive literature search was conducted across PubMed, Web of Science, and Scopus to identify relevant studies (2014–July 2026). Search terms combined “functional foods”, “polyphenols”, “probiotics”, “precision nutrition”, “Nrf2”, “oxidative stress”, “inflammation”, “autophagy”, “periodontitis”, “inflammatory bowel diseases”, “Alzheimer’s disease”, “oral-gut-brain axis”, “microplastics”, and “nanoplastics”. Both preclinical and clinical studies were included.

Specifically, database-specific search strategies were developed using Boolean operators and adapted to each database. All retrieved references were imported into Rayyan, which was used to facilitate screening procedures. Titles and abstracts were systematically examined, and studies clearly unrelated to the research questions were excluded at this stage. Potentially eligible articles underwent full-text assessment and were subsequently analyzed and included in the final narrative synthesis when considered relevant to the aims of the review.

To improve results, we also used the PRISMA flowchart as a framework for identifying, screening, assessing eligibility, and removing duplicate records. The resulting records were analyzed and subsequently incorporated into the manuscript.

The eligibility criteria included the following: (a) original research articles, review articles, and systematic reviews/meta-analyses; (b) in vitro studies, in vivo investigations, and human clinical trials focusing on precision nutrition approaches relevant to oral–gut–brain axis disorders, particularly periodontitis, inflammatory bowel disease, and Alzheimer’s disease; (c) studies addressing microplastics, nanoplastics, and their effects on human health; (d) investigations reporting molecular mechanisms and/or clinical outcomes; and (e) studies employing innovative models to explore the complex interactions between nutrients, the oral–gut–brain axis, and related disorders.

Studies were excluded if they (a) did not report relevant molecular mechanisms or outcomes related to microplastic or nanoplastic exposure, or (b) consisted of case reports or interventions not aligned with a precision nutrition approach.

Based on the criteria of high barrier permeability (oral–gut–blood–brain), efficacy against periodontal pathogens (e.g., *Porphyromonas gingivalis*), and modulation of the systemic inflammatory axis connecting to AD and IBD, the following food nutrients and natural compounds are supported by recent evidence (2019–2026) to be specifically linking these compounds to cellular stress resilience response through the modulation of the Nrf2 pathway. Nutritional compounds were selected for their local antioxidant and anti-inflammatory properties, useful for protecting the oral and intestinal barriers, which represent the primary site of interaction following the ingestion of MNPs. Finally, the selection of nutritional compounds took into account their ability to promote neuroprotection at the BBB level, thus alleviating oxidative stress and inflammation along the oral–gut–brain axis.

## 3. The Oral–Gut–Brain Axis

The oral microbiota is the second largest microbial community in humans, surpassed only by the gut. The oral cavity represents a highly complex microbial ecosystem that hosts more than 700 bacterial species, together with fungi, viruses, and archaea, organized within structured biofilm communities that occupy distinct ecological niches [21]. Although the oral cavity is anatomically connected to the gastrointestinal tract, the oral and gut microbiotas display markedly different microbial compositions [22]. This distinction is largely maintained by both anatomical and biochemical barriers, including the stomach and small intestine, as well as the antimicrobial effects of gastric acid and bile, which limit the direct transfer of microorganisms such as bacteria, fungi, protozoa, and viruses between the two environments [23]. The oral microbiota plays a crucial role in supporting physiological functions, including digestion, host defense against pathogens, and the maintenance of oral homeostasis. However, dysregulated epithelial responses can contribute to pathological states. Recent research has shown that flagellated oral bacteria trigger gingival epithelial cells to produce interleukin-23 (IL-23) via TLR5 receptors [24]. The gut microbiome consists of a diverse population of microorganisms that colonize the gastrointestinal tract, particularly the intestine. It is critically involved in maintaining host health through its roles in nutrient digestion and absorption, immune system regulation, and the biosynthesis of essential vitamins, neurotransmitters, and other bioactive compounds [25]. Nevertheless, oral microorganisms can reach the gastrointestinal tract, ultimately reaching the brain and influencing brain function, cognition, and host physiological and pathological pathways [26]. Recent findings from saliva and feces collected worldwide have shown that healthy intestines can be colonized by oral bacteria [21]. The primary oral bacteria phyla comprise *Firmicutes* (*Gemella*, *Granulicatella*, *Streptococcus*, and *Veillonella genera*), *Bacteroidetes* (strongly represented by *Prevotella*), *Proteobacteria* (*Neisseria* and *Haemophilus genera*), *Actinobacteria* (*Corynebacterium*, *Rothia*, and *Actinomyces genera*), and *Fusobacteria* (*Genus Fusobacterium*). These oral microorganisms exist in a symbiotic balance to aid digestion of food, resistance against pathogens, maintenance of homeostasis, and the modulation of the immune system, contributing to oral and general well-being. However, an imbalance (dysbiosis) in the oral microbiome is responsible for a variety of oral diseases [27].

### 3.1. Dysbiosis and Neurodegeneration

Compelling evidence reveals that the oral and gut microbiomes deeply influence the onset of neurodegenerative diseases, particularly AD, via the oral–gut–brain axis [28,29]. Periodontitis is a chronic multifactorial disease driven by dysbiotic polymicrobial communities and characterized by a destructive inflammatory response affecting the supporting structures of the teeth, resulting in tissue breakdown, alveolar bone loss, and impaired quality of life. Periodontal bacteria mainly including *Porphyromonas gingivalis*, *Tannerella forsythia*, *Treponema denticola*, *Campylobacter rectu*, and others, like *Prevotella intermedia*, *Fusobacterium nucleatum*, and *Aggregatibacter actinomycetemcomitans*, bypass compromised biological barriers, driving chronic inflammation and the mobilization of innate immune response-related signaling mediators (e.g., IL-1, IL-6, TNF-α), as well adaptive immunity mechanisms (expression of Th1, Th2, Th17, and Tregs) associated to a reduction in anti-inflammatory markers (IL 10) [30,31,32]. Chronic inflammation is known to play a pivotal role in the pathological process of both, serving as a connecting link between periodontitis and AD pathogenesis [33]. Under pathological conditions, oral microbiota can colonize the gut and disrupt the intestinal microbiota [34,35]. However, oral and intestinal inflammation related to gut microbiota dysbiosis has been considered an important feature in the progression of AD [36]. Recent evidence has shown that continuous gavage of periodontitis-related salivary microbiota impaired cognitive function and increased β-amyloid accumulation and neuroinflammation associated to gut microbial dysbiosis, intestinal pro-inflammatory responses, intestinal barrier impairment, and the subsequent exacerbation of systemic inflammation. This suggests that the periodontitis-related salivary microbiota may exacerbate AD pathogenesis in transgenic mice via the gut–brain axis [37]. Moreover, untreated periodontitis in patients with cognitive impairment may accelerate AD [38]. This occurs due a reduced ability to take care of oral hygiene and an increase in systemic pro-inflammatory cytokines, pathogens, and compromised BBB integrity [39]. Of note, post-mortem examination of brains from individuals with AD has confirmed the presence of periodontal pathogens, including *Actinomycetes* [40], *P. gingivalis* [41], *Helicobacter pylori* [42], and *Chlamydia pneumoniae* [43]. The salivary microbiota composition in patients with periodontitis shows greater microbial diversity and a significant overabundance of pathogenic bacteria compared to healthy individuals [44]. Specifically, patients with AD exhibit higher levels of *Fusobacteriota* and *Peptostreptococcaceae* and lower levels of *Veillonella* in saliva compared to the mild cognitive impairment (MCI) and control groups [45]. This postulates that the analysis of oral microbiota dysbiosis biomarkers in elderly subjects may represent a highly valuable, non-invasive method for identifying individuals at risk of AD and MCI.

### 3.2. Nutritional Modulation of the Oral–Gut–Brain Axis

The deterioration of oral health represents a key contributor to the development and progression of several systemic diseases and may also play an important role in accelerating biological aging and exacerbating age-related pathological conditions. Recent research confirms that targeted nutritional approaches are emerging as a promising, non-pharmacological strategy for managing AD or slowing its progression by modulating the oral–gut–brain axis. By using functional foods and probiotics, these approaches can restore microbial balance (dysbiosis) and mitigate the neuroinflammation, amyloid plaque formation, and tau protein aggregation associated with AD [46,47,48,49]. Dietary habits also play a pivotal role in shaping the composition and stability of oral microbial communities [47]. In particular, frequent consumption of sugars and refined carbohydrates favors the proliferation of cariogenic species, such as *Streptococcus mutans*, thereby increasing the risk of oral diseases [50]. In contrast, fermented dairy products enriched with probiotics, including *Lactobacillus rhamnosus* SD11, have been shown to enhance oral microbial diversity and to support a healthier oral ecosystem in humans [51]. Interestingly, while many dietary components exert beneficial effects on the gut microbiota, some may have divergent impacts on oral microbial communities. For example, fermentable carbohydrates such as fructo-oligosaccharides act as prebiotics by selectively promoting the growth of beneficial intestinal bacteria, including *Bifidobacterium* and *Lactobacillus* species. However, their fermentation in the oral cavity may also influence local microbial dynamics, highlighting the complex interactions between diet, oral health, and gut microbiota composition [52]. The selected studies allow us to speculate that an oral–gut–brain communication network exists, and bacteria likely reach the brain via trigeminal and vagus nerves. Mechanistically, the hematogenous pathway acts as a crucial, tri-directional communication in the oral–gut–brain axis. This important mechanistic framework implies that pathogenic oral bacteria; virulence factors, i.e., lipopolysaccharides (LPS) and gingipains; and inflammatory cytokines entering the bloodstream through disrupted oral mucosa reach the gut, trigger chronic systemic inflammation, and ultimately cross the BBB. This translocation and dissemination of oral bacteria into systemic circulation, often caused by periodontal disease (dysbiotic oral microbiota) and gut dysbiosis triggers a vicious cycle of chronic inflammation, creating a higher predisposition to the development of neuroinflammation and the progression of neurodegenerative diseases [53].

## 4. MNPs Promote Barrier Dysfunctions and Gut Microbiome Alterations

Environmental pollution by MNPs has reached alarming levels. The potential health risk associated with human exposure to these hazardous materials explains the keen interest in the topic shown by the world and the entire scientific community. The presence of plastics in various environmental matrices has also caused them to enter the food chain [54]. The involuntary ingestion of food contaminated with MNPs is the main route of human exposure to them [55]. The gut microbiota contributes to the development of immune defenses and the maintenance of the mucosal barrier, which is essential for the regulated absorption of nutrients and for preventing the entry of harmful agents. When this barrier is compromised, intestinal permeability increases, allowing the passage of damaging substances and microbial components. Furthermore, an imbalance in the microbial community can reduce biodiversity and promote the overgrowth of pathogenic microorganisms. The alteration of the Firmicutes/Bacteroidetes ratio is a potential indicator of alteration of the microbial flora. Numerous studies suggest that intestinal dysbiosis is associated with the onset of various pathological conditions, including IBD. The latter are disorders characterized by a chronic and remitting inflammatory condition of the intestinal tract, in which phases of exacerbation alternate with phases of remission. Its complex pathogenesis involves a multifaceted interplay between intestinal dysbiosis, environmental factors, genetic mutations and an abnormal immune response [56]. A common feature of IBD is the compromised intestinal barrier and the breakdown of tight junction proteins, which allows harmful bacteria, toxins, and environmental factors to leak into the underlying tissue. This triggers an aggressive immune response and chronic inflammation, which ultimately drives tissue ulceration. In healthy conditions, the intestinal barrier protects the mucosa from negative influences of the intestinal lumen through a thick layer of mucus that avoids direct contact with bacteria, tight connections in the epithelium to block bacterial infiltration, and antimicrobial peptides (AMPs). Also important are the processes of autophagy, which offer antibacterial defense to the host, also through the degradation of invading bacteria and the secretion of IgA, which influences the efficiency of the immune system in keeping intestinal bacteria under control. In subjects affected by IBD, the protective mechanisms of the mucosal barrier are altered. The reduction in the mucus layer and the production of AMP, the alteration of autophagy processes and the enhance in the permeability of the IEB no longer guarantee adequate protection of the epithelium from the adhesion and invasion of luminal bacteria. This is followed by excessive activation of the host’s compensatory immune reactions, which trigger chronic intestinal inflammation [57].

### MNPs, Intestinal Barrier Integrity and Inflammatory Bowel Disease

The correlation between MNPs ingestion, intestinal dysbiosis and IBD has been widely discussed [58,59]. The composition of the intestinal microbiota in subjects who have developed chronic intestinal inflammatory diseases after exposure to microplastics is significantly modified [60]. In general, a depletion in the abundance of Firmicutes and Bacteroidetes and an increase in Proteobacteria have been observed [35,61]. Compared to healthy controls, IBD subjects were found to have reduced levels of *Lachnospiraceae*, *Akkermansia muciniphila* [62], and the butyrate-producing species *Roseburia hominis*, *Faecalibacterium prausnitzii*, and *Eubacterium rectale* [63]. The abundance of beneficial bacteria, such as *Lactobacillus* and *Bacteroides*, is also attenuated, causing oxidative stress, intestinal dysbiosis, and inflammation [64]. On the contrary, higher levels of *Ruminococcus torques* and *Ruminococcus gnavus* [65,66,67] and sulfate-reducing bacteria such as *Desulfovibrio* were highlighted. The harmful action exerted by the hydrogen sulfate produced damages to the epithelial cells, causing inflammation of the mucosa [68]. An increase in *Veillonella* spp., *Actinomyces* spp., and *Coprococcus* spp. in patients with IBD and in *Intestinibacter* spp. in subjects affected by both chronic inflammatory intestinal forms has also been reported [69]. In addition, in subjects suffering from IBD, there is also an increase in invasive-adherent *Escherichia coli*, which damages the epithelium of the intestinal mucosa. The consequent increase in intestinal permeability allows the passage of metabolic waste and bacterial toxins, such as lipopolysaccharide, into the blood, exacerbating the inflammatory condition [70,71,72]. The production of superantigens by *Staphylococcus aureus* also appears to aggravate the inflammatory process [73]. Li et al. [74] and Chen et al. [75] demonstrated the correlation between the alteration in the intestinal microbiota and the onset of IBD in mice and the increase in Staphylococcus aureus following exposure to PE and PVC, respectively. An interesting study conducted by Fournier et al. [76] showed how the ingestion of PE microparticles largely modified the composition of the human intestinal microbiota. In detail, an increase in potentially harmful pathobionts, such as *Desulfovibrionaceae* and *Enterobacteriaceae*, and a concomitant decrease in beneficial bacteria, like *Christensenellaceae* and *Akkermansiaceae*, have also been documented. Furthermore, Ghosal et al. [60] showed the correlation between the increase in *Desulfovibrio* and the increased amount of indole and 3-methylindole in the intestine of patients with IBD following exposure to PE particles. Notably, 3-methylindole is an organic compound that derives from the decarboxylation of the amino acid tryptophan by the intestinal microbiota. Since this compound is not usually present in the large intestine of healthy individuals, it is possible to deduce that its altered production following exposure to PE is associated with intestinal dysbiosis induced by the ingestion of plastics. Ghosal also corroborated the results of the study conducted by Tamargo et al. [77], in which an increase in *Proteobacteria* and *Synergistetes* was detected following the ingestion of PET microparticles. The results suggest the use of these two bacterial populations as biomarkers of exposure to MPs in the human intestinal microbiota. Furthermore, following the effects of exposure to PE microparticles in an in vitro intestinal model of an adult human being, an increase in intestinal pathobionts (*Desulfovibrionaceae*, *Dethiosulfovibrionaceae* and *Enterobacteriaceae*) and a reduction in bacterial populations indicative of good intestinal health, such as *Akkermansiaceae* and *Christensenellaceae*, have been also observed. 

## 5. MNP-Driven Inflammation and Oral–Gut–Brain Axis Disorders

MNPs act as environmental pollutants and vectors for toxic chemicals by initiating localized inflammation in the oral cavity and gut, which subsequently propagates to the brain via the oral–gut–brain axis [78]. Ingested/inhaled MNPs from food, water, or dental materials cause oral mucosal irritation, oral dysbiosis (imbalance in bacteria), and damage to gingival fibroblasts. This can accelerate periodontal disease, which is linked to neuroinflammation. MNPs reach the intestine, damaging the IEB (“leaky gut”) and altering the microbiota, promoting the growth of harmful bacteria (e.g., *Enterobacteriaceae*) [79]. Specifically, MNP exposure can compromise this barrier by inducing ROS-mediated apoptosis in epithelial cells, thereby increasing permeability in the duodenum, jejunum, ileum, and colon [80]. Experimental in vivo studies indicate that MP-induced gut dysbiosis can stimulate the expression of pro-inflammatory cytokines, e.g., IL-1β, TNF-α, IL-6, TLR-4, activator protein 1 (AP-1), and interferon regulatory factor 5 (IRF5), which further aggravate barrier damage and perpetuate intestinal inflammation [81,82].

### 5.1. MNP Oral and Intestinal Inflammation

**Figure 1:**
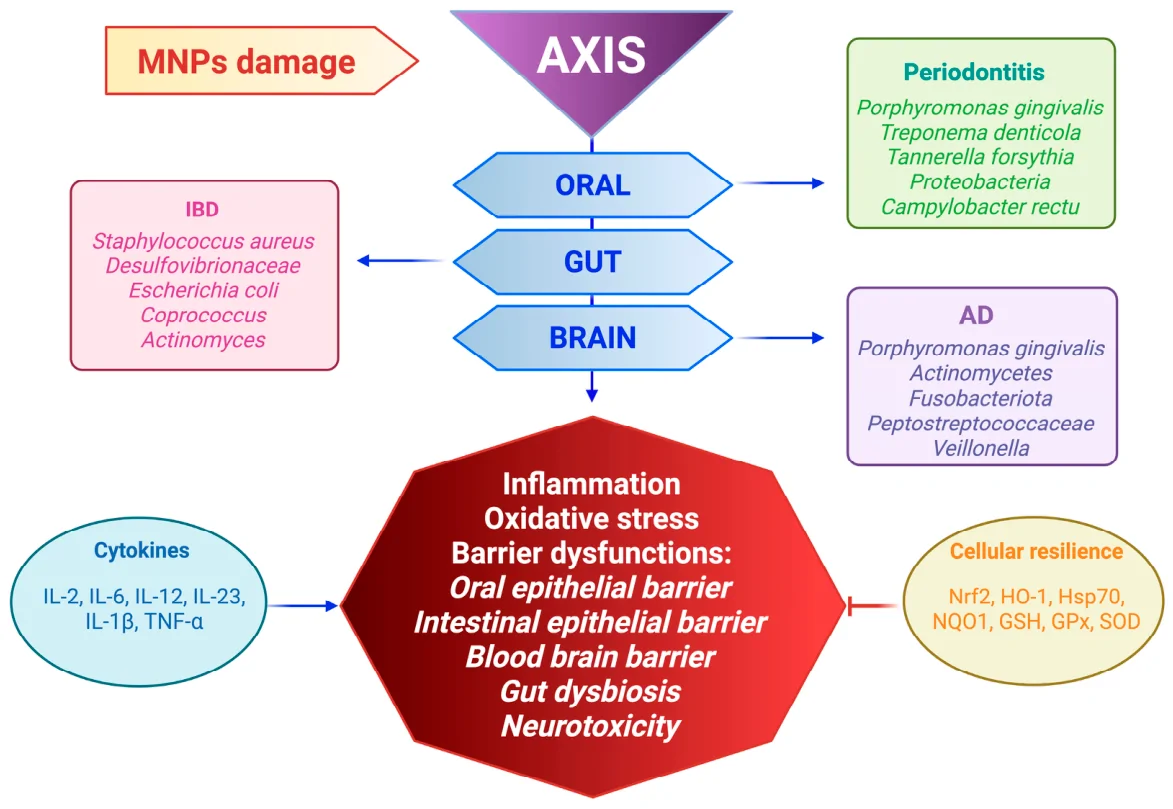
Schematic representation of the damage caused by MNPs. Created in BioRender. https://BioRender.com/2xpl3ht (accessed on 21 July 2026). The blue arrow indicates activation 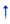, while the red arrow indicates inhibition 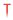.

Inflammation from the gut spreads systemically, leading to neuroinflammation that may accelerate neurodegenerative disorders [81]. Nanoscale MNPs can directly cross the BBB through endocytosis, pinocytosis, or the “Trojan horse” mechanism (transported by immune cells), and they can accumulate in key regions like the hippocampus and cortex, causing neurotoxic effects, including protein misfolding and memory and cognitive deficits [83]. This pathological mechanism is driven by oral pathogens, such as *Porphyromonas gingivalis* (*P. gingivalis*), can travel from the mouth, survive the gastric environment, and be transported by immune cells (macrophages/dendritic cells) to the gut, where they further exacerbate systemic inflammation that subsequently reaches the brain (neuroinflammation), disrupting the oral–gut–brain axis [84]. Firstly, MNPs can interact with periodontopathic bacteria in various ways. At the molecular level, MNPs can serve as a “colonization substrate” for biofilm-producing bacteria, thereby acting as a vector [85]. Secondly, MNPs are known to modulate microbial behavior by enhancing their virulence and resistance to host immune responses. For instance, *P. gingivalis* has been shown to attach to MPs and develop biofilms, which shield the bacterium from immune surveillance (macrophage phagocytosis) by host organisms and even from antimicrobial therapies, ultimately increasing overall virulence. MNPs not only promote the growth of microbes but also cause a shift in oral microbiota composition, favoring the growth of pathogenic bacteria and/or suppressing beneficial commensals species [85]. Recent research has shown that MNPs are able to cross the gut barrier in mice and zebrafish, and they can subsequently cross the BBB via systemic circulation, ultimately promoting neurotoxicity in the central nervous system (CNS) [9,86]. Figure 1 provides a schematic overview of MNP-induced oral–gut–brain axis dysfunction.

### 5.2. Periodontitis and Oral Dysbiosis

Periodontitis is an oral pathology caused by an inflammatory response affecting the periodontal tissues, particularly alveolar bone, cement, gingiva, and periodontal ligament. Recently, growing evidence has shown the close association between periodontitis and the onset of gastrointestinal and brain disorders caused by oral infections [87]. The correlation between periodontitis and AD is a topic of growing scientific interest; however, the available evidence is still limited and is mainly based on a small number of preclinical studies, which suggests a possible involvement of common inflammatory, immune, and microbiological mechanisms in the pathogenesis of both pathologies. Although there is limited evidence and available results are variable, researchers suspect a bidirectional relationship between AD and periodontal disease [88,89]. The underlying biological mechanism may be chronic neuroinflammation caused by *P. gingivalis*, a bacterium responsible for periodontitis. This bacterium has the ability to release virulence factors (e.g., gingipains, amyloidogenesis, and glial cell activation) that enter the brain via the bloodstream, neural pathways, or the oral–gut–brain axis [90,91]. The research by Dominy et al. identified *P. gingivalis* DNA and its toxic proteases (gingipains) in the brain and cerebrospinal fluid (CSF) of AD patients, suggesting that the detection of *P. gingivalis* DNA in the CSF could be a potential diagnostic marker for AD. Furthermore, the same authors found that exposure to gingipains, a major virulence factor of *P. gingivalis*, increased the degeneration of neurons by triggering NLRP3 inflammasome activation, resulting in caspase-1 activation and neuronal pyroptosis. This in turn leads to the release of a severe neuroinflammatory cascade (IL-1β and IL-18), which accelerates both neuronal death and Aβ plaque formation in AD-affected brains compared to the control group. In particular, after 6 weeks of exposure to *P. gingivalis*, Aβ1–42 levels significantly increased in the mouse brain. Given this, researchers suggested inhibiting gingipain might be beneficial for AD [90]. Similarly, in the study performed by Wu et al., mice exposed to 1 mg/kg daily lipopolysaccharide from *P. gingivalis* 5 weeks in a row showed significantly increased microglial IL-1β expression, as well as memory and learning deficits, besides the accumulation of Aβ in neurons [92]. These findings are highly significant for clinical practice, as studies show that individuals with AD and dementia exhibit poorer oral health (caries and periodontal disease) compared to cognitively healthy peers, and that this condition worsens the quality and quantity of the saliva [93]. A recent clinical study performed in an elderly cohort of 468 participants observed that microbiological and host response features of periodontitis were linked to adverse MRI markers typical of AD, highlighting, in particular, the association between greater periodontitis extent and lower entorhinal cortex volume and cortical thickness in brain regions implicated in AD [94].

#### MNPs and Periodontal Inflammation

Mounting evidence indicates that environmental pollutants and lifestyle factors significantly increase periodontitis risk by inducing systemic oxidative stress and oral microbiome dysbiosis [14]. Indeed, a study by Han et al. using gingival fibroblasts exposed to PET-MPs (50–100 µg/mL) showed dose-dependent cytotoxicity, oxidative stress induction, and pro-inflammatory activation. Specifically, PET-MP exposure upregulated pro-inflammatory signaling markers including caspase-3, IL-1β, TNF-α, iNOS, and PGE-2, while it downregulated AKT1 and ALPL genes, exacerbating periodontal pathogenesis [95]. Nutritional strategies with natural constructs targeting the Nrf2 pathway can exert therapeutic potential in treating periodontitis and enhancing the regeneration of periodontal tissues [96]. Accordingly, a nanotherapeutic system (e.g., HP-PVA@MH/Fe-Que) modulates the Nrf2/NF-κB signaling to effectively scavenge ROS, polarize macrophages toward an IL-4-driven anti-inflammatory M2 phenotype, reduce inflammation, and ultimately enhance the osteogenic differentiation of human periodontal ligament stem cells (hPDLSCs) in periodontal tissues. This dual-action mechanism actively heals inflamed tissue by suppressing tissue-damaging inflammatory signals (IL-1β and TNF-α) and increasing anti-inflammatory cytokine expression (IL-10 and Arg-1) as well as the antioxidant defense system (SOD-1 and CAT). Finally, even in rats with periodontitis, the HP-PVA@MH/Fe-Que hydrogel can effectively scavenge ROS, mitigate inflammation, restore periodontal homeostasis, and promote alveolar bone repair [96].

### 5.3. MNP Exposure and Inflammatory Bowel Disease Susceptibility

Inflammatory bowel diseases (IBDs), including Crohn’s disease and ulcerative colitis, are chronic conditions mainly caused due to altered mucosal immune responses to the gut microbiome in genetically predisposed individuals [97]. The potential correlation between inflammatory bowel disease (IBD) and exposure to MNPs is a topic of growing scientific interest; however, available knowledge is still preliminary and based primarily on a limited number of epidemiological and experimental studies, which indicate that MNPs may contribute to intestinal inflammation, microbiota dysbiosis, and impaired intestinal barrier function—key factors in the pathogenesis of IBD. Furthermore, the interpretation of results is currently limited by the marked heterogeneity of available studies, which differ in terms of the type of polymer used, particle size and shape, dosages employed, exposure times, and experimental models adopted. The lack of standardized protocols therefore makes direct comparisons between studies difficult and limits the ability to draw definitive conclusions on the role of MNPs in the development and progression of IBD. Recently, it has been widely believed that environmental pollutants, detected in human tissues, contribute to the development of IBD [15,56]. An epidemiological study found the patients with IBD have approximately 50% higher concentrations of fecal MPs than healthy individuals [15]; however, the underlying molecular mechanisms remain unclear.

#### Mechanisms of MNP-Induced Intestinal Injury

Recent studies have shown that the uptake of three different types of NPs in both caco-2 cells and in mice increased in a time- and concentration-dependent manner. The toxicity of MNPs is closely related to their particle size, dose, and shape. Generally, smaller-sized particles, particularly PS-NPs PS-COOH and PS-NH_2_, exhibited a higher internalization and more efficient cellular uptake rates than pristine and larger-sized MPs [6]. Among small particles, PS-NH_2_, due to their positively charged surface, are more toxic than carboxylated or unmodified particles. In caco-2 cells, PS-NH_2_ disrupts cell membranes and mitochondria as well as induces apoptosis, primarily through internalization by macropinocytosis and clathrin-mediated endocytosis [6]. Conversely, larger particles (e.g., PS-MPs), though less readily internalized, are more likely to cause mitochondrial membrane damage in colon epithelial cell models than smaller PS-NPs [98]. Intriguingly, the exposure to PS-NH_2_ NPs at a higher concentration of 120 μg/mL significantly increased the rates of necrotic cells with the activation of RIPK3/MLKL-mediated necroptosis. Similarly, PS-NH_2_ NP exposure increased expression of IL-1β and TNF-α in the ileal lamina propria, causing a severe intestinal inflammation in mice [99]. These data confirmed that MP exposure tends to trigger the necroptosis of intestinal epithelial cells rather than apoptosis, both in vivo and in vitro. Furthermore, animal studies have observed that MNPs accumulate primarily in the digested tract, which can induce gut microbiota dysbiosis, inflammation, oxidative stress, and increased intestinal permeability [4,100,101,102]. Recent research in mammalian models revealed that systemic toxicity induced by MPs in different organs is highly dependent on particle size, with smaller particles demonstrating greater biodistribution. Specifically, smaller MPs (0.5 µm) easily translocated and heavily accumulated, inducing more serious inflammation and mechanical damage in the spleen, kidney, heart, lung, and liver. However, larger MPs (5 µm) led to more severe intestinal barrier dysfunction, as well as gut dysbiosis and metabolic disorder in association with neuroinflammation [4]. Mechanistically, PS-MS treatment alone does not directly induce colon inflammation in healthy mice; however, PS-MS at a dose of 10 mg/kg and 5 µm of diameter suppresses intestinal Muc2 protein expression and impairs the mucus barrier by reducing the abundance of *Lachnospiraceae* NK4A136 group and butyrate levels, thereby inhibiting PPARγ signaling and exacerbating colitis [103]. Treatment with exogenous sodium butyrate supplementation restores mucosal homeostasis via PPARγ activation [103].

### 5.4. MNP Exposure and Alzheimer’s Disease Risk

AD is the most prevalent neurological disorder, affecting more than 7 million Americans and 30 million people worldwide [104]. This number could grow to 13.8 million by 2060. In Italy, it is estimated that approximately 1.4 million people live with dementia, 50–60% of whom suffer from AD (around 600,000 individuals) [105].

The potential correlation between MNP exposure and AD is an emerging area of research. However, currently available evidence is still limited and derives primarily from preclinical and experimental studies, which suggest a possible involvement of MNPs in neuroinflammation, oxidative stress, blood–brain barrier dysfunction, and the accumulation of neurotoxic proteins associated with neurodegeneration. Furthermore, the heterogeneity of experimental models, types of polymers used, particle sizes, doses, and exposure times limits the comparability of results and makes it difficult to extrapolate definitive conclusions on the effects of MNPs on the development and progression of AD in humans.

Emerging research supports the “oral–gut–brain axis” hypothesis, linking chronic periodontitis and specific pathogens like *P. gingivalis* to the pathogenesis of AD [90]. MNPs may exacerbate periodontitis and promote the entry of *P. gingivalis* or its neurotoxic proteases (e.g., gingipains) into the brain. This systemic cascade accelerates neuroinflammation and the formation of amyloid plaques, acting as a contributing risk factor for AD [106]. MNPs have been detected in various regions of the human body, including blood, organs, placenta, gastrointestinal system, and brain, establishing a worrying association with serious health complications such as obesity, diabetes, cognitive impairment, and AD [106,107]. A significant concern is the ability of MNPs to cross the BBB, potentially contributing to neurotoxicity, neuroinflammation, and dementia. To date, our understanding of the brain impact and the pathophysiology/pathways involved in MNP-related adverse health consequences is limited; thus, it is crucial to further investigate how plastic particles can impact brain function, using systematic methods to assess the risks associated with these mental health conditions [108].

#### Mechanisms of MNP-Induced Neurotoxicity

Recent data have shown that orally ingested NPs (30–50 nm) can accumulate in the brains of adult mice, causing cognitive decline. The preferential uptake of NPs triggers a neurotoxic cascade leading to microglial activation, dysregulation of hippocampal neuronal activity, and induction of inflammatory responses [109]. Results from a recent in vitro study demonstrated that although low-dose PS-NPs do not exhibit obvious neurotoxicity, they significantly accelerate the nucleation rate of Aβ40 and Aβ42 peptides, prompting heightened Aβ oligomers. This synergistic combination between PS-NPs and low-concentration Aβ oligomers exacerbates neurotoxicity, ultimately leading to severe cell membrane damage, mitochondrial dysfunction, and elevated intracellular ROS and Ca^2+^ levels [107]. Detailed research is required that expands the scope of human studies, particularly regarding MNP exposure levels, their influence on gut microbiome and cerebral function, along with comprehensive clinical evaluations to translate animal-model pathology to human health [110]. Taken together, the above data have established that MNPs can trigger neurodegeneration in the brain, but the precise underlying mechanisms remain elusive. Furthermore, most current research is based on animal models or in vitro cellular experiments, and the exposure doses and modes of MNPs used in experiments may differ from real-world scenarios. This discrepancy represents a major hurdle in the current research on environmental pollutants, resulting in a critical translational gap between laboratory settings and actual human exposure. Therefore, it is necessary to carefully simulate exposure conditions encountered in real environments, including the exposure dose, duration, and molecular pathways involved, to assess the potential risks of MNPs on the CNS disorders.

## 6. Functional Foods, Nrf2 Signaling, and Cellular Resilience: Emerging Evidence for Oral–Gut–Brain Axis Health

**Figure 2:**
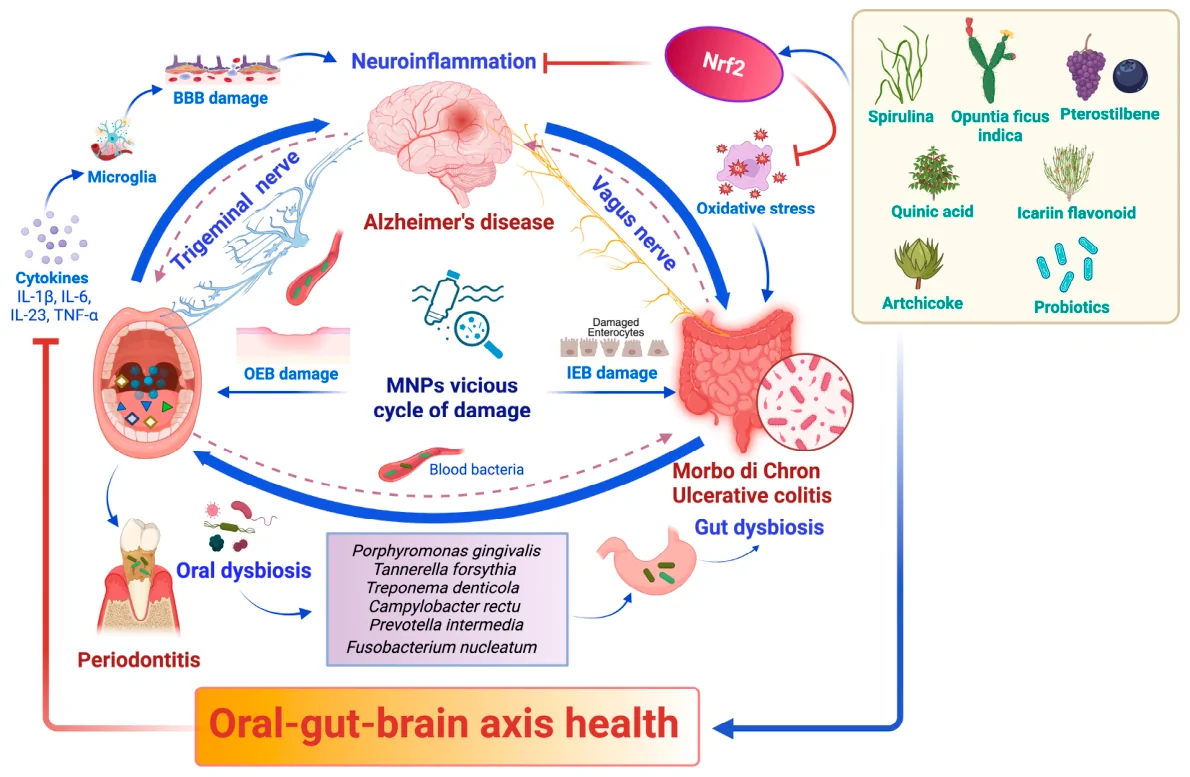
Schematic representation of the bidirectional role of the oral–gut–brain axis modulated by functional foods targeting the Nrf2 pathway. Created in BioRender. https://BioRender.com/5gl6zkm (accessed on 20 July 2026). The blue arrow indicates activation 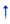, while the red arrow indicates inhibition 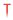. The solid blue arrow indicates direct oral-gut interaction, whereas the dashed purple arrow represents hematogenous dissemination through the bloodstream.

Functional foods, including artichoke, quinic acid, *Opuntia ficus-indica*, spirulina, pterostilbene, and icariin, contain bioactive compounds, i.e., flavonoids, polyphenols, alkaloids, terpenoids, and carotenoids, that when combined with probiotics offer significant preventive and therapeutic benefits. Emerging evidence has shown that these nutrients target Nrf2 cellular resilience signaling and could potentially mitigate oral–gut–brain axis disorders [111,112]. In summary, Figure 2 illustrates the role of functional foods in attenuating MNP-induced oral–gut–brain axis dysfunction via Nrf2 activation.

### 6.1. Artichoke

#### 6.1.1. Preclinical Evidence on Artichoke

Artichoke, a member of the Asteraceae family, has traditionally been used to counteract oxidative stress and inflammation associated with chronic diseases. Experimental evidence reported that a high dose (1.6 g) of artichoke methanolic leaf extract supplementation prevented diethylnitrosamine-induced oxidative damage, apoptosis, and neurotoxicity via the upregulation of the Klotho/PPARγ signaling pathway in BALB/c mouse brains after 14 days [113]. Furthermore, the anti-inflammatory effects of functional food intake, including artichoke, caihua, and fenugreek vegetal extract original blend (ACFB), on reducing the p65NF-κB pathway, COX-2 and PGE2 expressions, and IL-8 secretion in caco-2 epithelial cells have been investigated [114]. Moreover, *Cynara cardunculus* L. attenuated the TNF-α-induced activation of the NF-κB signaling pathway and reduced the expression of the pro-inflammatory mediators IL-8 and COX-2 associated with an upregulation of the Nrf2 pathway and resilience genes stimulating an adaptive response in vitro [115]. In addition, an interesting study observed that cynaropicrin, a sesquiterpene lactone, exhibits preventive and pharmacological effects in periodontal diseases. Specifically, it suppressed the bacterium *Porphyromonas gingivalis*-induced NF-κB pathway and IL-8 and IL-6 cytokine synthesis in human gingival fibroblasts in a dose-dependent manner via the inhibition of RANKL-induced osteoclast differentiation [116]. Moreover, Chen et al. demonstrated that cynarin exerts protective effects against ulcerative colitis, both in vitro and in vivo, by suppressing STAT3/NF-κB signaling and limiting the polarization of macrophages toward the pro-inflammatory M1 phenotype [117]. Lastly, another important study conducted by Deng and coworkers revealed that artichoke water extract at 0.8 and 1.6 g/kg, in particular, the most abundant flavonoids including chlorogenic acid and cynarin, significantly reduced tissue Pb accumulation, induced fecal Pb excretion, improved lipid profiles, and attenuated liver injury by activating Nrf2 antioxidant signaling and stress resilience enzymes SOD2, CAT, and GPX4 and inhibiting MDA and 8-OHdG in mice [118]. Similarly, a dose of 0.8 and 1.6 mg of artichoke water extract, targeting the activation of Nrf2 signaling, blocked NLRP3/caspase-1/GSDMD-mediated pyroptosis in HepG2 cells [118].

#### 6.1.2. Clinical Evidence on Artichoke

Clinical evidence for artichoke includes a randomized, double-blind, placebo-controlled trial demonstrating that very-long-chain inulin extracted from globe artichoke significantly increased fecal populations of bifidobacteria and lactobacilli and favorably modulated gut microbiota composition, highlighting the contribution of artichoke in regulating the gut–brain axis through prebiotic mechanisms [119]. Table 1 provides an overview of the effects of artichoke on the modulation of the main molecular pathways discussed in this review (↓ inhibition or ↑ activation).

### 6.2. Quinic Acid

#### 6.2.1. Preclinical Evidence on Quinic Acid

Quinic acid (QA), a naturally occurring cyclohexanecarboxylic acid present in various plant species, has been reported to possess a broad range of biological activities, including antibacterial, antioxidant, anti-inflammatory, and antiviral properties [144]. A recent study observed that 30 mg of QA attenuated brain oxidative stress by reducing malondialdehyde and increasing levels of stress resilience markers such as SOD, CAT, and glutathione peroxidase (GPx), and it suppressed neuroinflammatory cytokines induced by high-fat diets and improved gut microbiota dysbiosis by decreasing the *Firmicutes/Bacteroidetes* ratio [120]. In addition, the results indicated that mice treated with QA significantly altered the composition of metabolites in the feces. Specifically, QA increased the relative abundance of tryptophan, indole-3-acetic acid (IAA), and kynurenic acid [120]. Interestingly, QA treatment promoted hippocampal neurogenesis by proliferating and differentiating neural progenitor cells and recovering neurons from stress caused by oxygen glucose deprivation [121], targeting the Notch pathway in vitro and in vivo [122]. Moreover, a dose of 60 and 100 mg/kg of QA has shown antioxidant potential through its ability to activate the Nrf2 pathway and its downstream target genes in rat models of ulcerative colitis [145]. The bioactive flavonoids, particularly QA from *Eucommia ulmoides* leaves, attenuated cognitive dysfunction by regulating the JNK/TLR4 signaling pathway in DSS-induced colitis in mice [124]. Of note, QA and its amide derivatives (2–4) reduced phytohemagglutinin (PHA)-induced neurotoxicity by inhibiting p38 MAPK activation in SH-SY5Y cells [125]. In addition, the chronic administration of 5-caffeoylquinic acid attenuated Aβ deposition in the brain through the regulation of Aβ clearance pathways and was associated with reduced cognitive impairment and neuronal loss in APP/PS2 mice [126]. Thus, it may be effective in preventing cognitive dysfunction in AD. Finally, computational studies using machine learning methods discovered and predicted that the bioactive compounds of *Centella asiatica*, dicaffeoylquinic acids, can protect against Aβ-toxicity in human neuroblastoma MC65 cells [146].

#### 6.2.2. Clinical Perspectives on Quinic Acid

The available knowledge comes mainly from preclinical models, while there are currently no clinical trials evaluating their efficacy in inflammatory bowel disease, periodontitis, Alzheimer’s disease, oral–gut–brain axis disorders, or toxicity associated with micro- and nanoplastics. Table 1 provides an overview of the effects of quinic acid on the modulation of the main molecular pathways discussed in this review (↓ inhibition or ↑ activation).

### 6.3. Opuntia ficus-indica

#### 6.3.1. Preclinical Evidence on *Opuntia ficus-indica*

The cladodes, fruits, and seeds of *Opuntia ficus-indica* (OFI) are known to be rich in polyphenols, flavonoids (especially isorhamnetin derivatives), betalains, and polysaccharides, which together contribute to its strong antioxidant and anti-inflammatory properties. OFI has therefore shown promise as a dietary supplement for conditions characterized by oxidative stress, chronic inflammation, and deregulated immune responses. In periodontal disease, advanced biomaterials have provided evidence for the role of OFI. A thermo-responsive hydrogel embedded with chitosan nanoparticles loaded with OFI extract has been developed for the local treatment of periodontitis [147]. In vitro, this system showed strong anti-biofilm activity against *P. gingivalis*, *S. mutans*, and *P. aeruginosa*, important pathogens of periodontal dysbiosis. Beyond its antimicrobial activity, the formulation enhanced the polarization of macrophages towards the anti-inflammatory M2 phenotype and reduced the release of pro-inflammatory cytokines. These immunomodulatory effects were mechanistically supported by the upregulation of antioxidant enzymes such as SOD and catalase, while Nrf2-mediated transcriptional activity was restored. Importantly, the hydrogel enabled controlled release, overcoming bioavailability issues that remain a challenge for the translational applications of dietary supplements. The aqueous cladode extract of OFI has recently been shown to exert strong antioxidant and anti-inflammatory effects both in vitro and in vivo. Rich in gallic acid, catechin, caffeic acid, and ferulic acid, it inhibits neutrophil-derived ROS and myeloperoxidase activity while limiting degranulation. In a rat model of acetic acid-induced ulcerative colitis, oral pretreatment with OFI extract protected against mucosal damage; preserved goblet cells; restored catalase, superoxide dismutase, and glutathione levels; and reduced plasma levels of the pro-inflammatory cytokines TNF-α and IL-1β. These results emphasize that OFI cladodes can counteract intestinal inflammation by attenuating neutrophil-induced oxidative stress and promoting redox balance, suggesting that they can be used as a food source for the treatment of ulcerative colitis [127]. OFI has been shown to activate Nrf2/SOD2 signaling, improve insulin sensitivity, and suppress NF-κB-mediated inflammation, while remodeling the gut microbiota towards anti-inflammatory taxa in obesity-associated colitis [128]. A similar anti-inflammatory profile mediated by Nrf2 activation and NF-κB inhibition has been observed in human hypertrophic adipocytes, which are characteristic of obesity [129]. Although OFI extracts have been extensively characterized and contain isorhamnetin, quercetin, kaempferol, ferulic acid, and the related derivatives [147], the exact bioactive constituents responsible for the anti-inflammatory effects and Nrf2 activation are not yet fully understood. Future studies should prioritize the use of standardized extracts and a thorough evaluation of their pharmacokinetic and pharmacodynamic properties in optimized pharmaceutical formulations.

#### 6.3.2. Clinical Evidence on *Opuntia ficus-indica*

A clinical study conducted in healthy subjects has demonstrated that the oral intake of OFI fruits rich in indicaxanthin (IX) is able to modulate the activity of intracortical glutamatergic circuits and neuronal plasticity processes, providing the first evidence in humans of a potential neuromodulatory action of this bioactive compound [148]. Another clinical study demonstrated that supplementation with OFI (1500 mg/day for 3 months) significantly increased total antioxidant capacity and reduced several biomarkers of oxidative stress, suggesting the role of this compound in the prevention of conditions associated with inflammation and oxidative damage [149]. Moreover, a randomized, double-blind, placebo-controlled clinical study demonstrated that supplementation with OFI extract for 8 weeks improved gut microbiota composition and gastrointestinal symptoms in subjects with intestinal dysbiosis, supporting its potential role in gut–brain axis-related diseases [150]. A randomized, placebo-controlled clinical study demonstrated that a synbiotic formulation containing Opuntia humifusa extracts significantly improved gastrointestinal symptoms and psychological well-being in elderly patients with irritable bowel syndrome, supporting modulation in the gut–brain axis [151]. The available human evidence includes two randomized placebo-controlled clinical trials and additional controlled clinical studies that have evaluated the effects of Opuntia on gastrointestinal health, gut microbiota composition, oxidative stress status, and the neurological potential of these compounds in disorders of the gut–brain axis. Table 1 provides an overview of the effects of *Opuntia ficus-indica* on the modulation of the main molecular pathways discussed in this review (↓ inhibition or ↑ activation).

### 6.4. Spirulina (Arthrospira platensis)

#### 6.4.1. Preclinical Evidence on Spirulina

*Arthrospira platensis*, commonly known as spirulina (SP), is a filamentous cyanobacterium known for its exceptional therapeutic potential for oral–gut–brain axis disorders [152,153,154]. It has a rich nutritional profile including bioactive peptides, phenolic compounds, and polyunsaturated fatty acids, which have powerful antioxidant, anti-inflammatory, and immunomodulatory properties. Over the last decade, SP has become increasingly recognized as a promising dietary supplement for the treatment of chronic diseases in which oxidative stress and inflammatory dysregulation play a central role. Recent preclinical evidence suggested that 12% SP hydrogel was found to have maximum antimicrobial efficacy against major periodontal pathogens, namely, *A. actinomycetemcomitans*, *P. gingivalis*, *T. forsythia*, *Prevotella Intermedia*, and *F. nucleatum* [155]. Evidence from clinical studies indicates that subgingival administration of a 4% spirulina gel may serve as an effective adjunct to non-surgical periodontal treatment, representing a natural and potentially more economical alternative to conventional antimicrobial agents such as chlorhexidine [156]. Both interventions improved probing depth, clinical attachment levels, and the microbial load of *Porphyromonas gingivalis*, with spirulina showing comparable efficacy to chlorhexidine without the side effects associated with prolonged antiseptic use. Taken together, these clinical studies provide the first translational evidence that spirulina can improve periodontal outcomes through anti-inflammatory and antimicrobial mechanisms, making it a valuable adjunct to conventional surgical therapy. The role of spirulina in intestinal inflammation and the homeostasis of the microbiota is the subject of intensive research. Preclinical studies with DSS-induced colitis models have shown that spirulina-based therapeutics alone or in combination with probiotics can significantly reduce intestinal inflammation, restore mucosal barrier integrity, and rebalance the gut microbiota while reducing IBD-associated psychiatric disorders such as anxiety and depression. For example, a spirulina-based cerium oxide nanocomposite alleviated colitis by scavenging ROS, downregulating pro-inflammatory cytokines (TNF-α and IL-6), and favoring the proliferation of anti-inflammatory bacterial taxa, namely, *Lachnoclostridium*, *Eubacterium siraeum*, and *Rikenella*, which produce short-chain fatty acids that promote intestinal epithelial cell proliferation, enhance intestinal barrier integrity, and exert anti-inflammatory effects [130,157,158,159,160,161]. Recent advances also include nanozyme-based delivery systems. Chen et al. developed an orally administrable spirulina–selenium nanoparticle complex that exhibited cascade-like ROS scavenging activity, suppressed pro-inflammatory cytokines, enhanced IL-10, and restored tight junction integrity in DSS-induced colitis [130]. This approach emphasizes how spirulina in combination with nanozyme technology can enhance antioxidant and barrier-protective effects in IBD. Beyond the effect on the balance of the gut microbiota, the effects of spirulina converge in combating oxidative stress in the colon through ROS neutralization, reduction in the overexpression of pro-inflammatory cytokines (TNF-α, IL-1β, and IL-6) in the colon, and the Nrf2-dependent antioxidant gene activation and inhibition of NF-κB signaling. The potential neuroprotective effects of spirulina are gaining increasing attention in the context of AD. In a sporadic AD mouse model induced by intracerebroventricular streptozotocin, spirulina extract prevented deficits in working, recognition, and spatial memory, while maintaining glutathione levels in the hippocampus and cortex. Treatment reduced lipid peroxidation, nitrite accumulation, and acetylcholinesterase activity while decreasing astrogliosis and microgliosis, indicating the suppression of neuroinflammation. Mechanistically, these effects have been attributed to the antioxidant activity of phycocyanin and polyphenols, which can scavenge free radicals and improve neuron survival [153]. These effects are consistent with evidence that phycocyanin enhances insulin receptor signaling and activates the PI3K/Akt pathway, thereby improving neuronal survival and counteracting tau hyperphosphorylation. Taken together, these results indicate that spirulina is a candidate for integrative strategies in AD, although large-scale clinical validation is still pending. Although direct evidence in oral–gut–brain disorders is still limited, studies in other contexts consistently show that spirulina exerts protection through Nrf2 activation and the induction of antioxidant genes [162]. Examples include attenuation of oral and gut toxicity [163,164], improvement of neurodegeneration [165], preservation of brain reserve [166,167], enhancement of mitochondrial biogenesis [168], and reduction in chronic gastrointestinal inflammation [169]. These findings indirectly support Nrf2 as a central axis for spirulina’s potential benefits in oral–gut–brain disorders. Recent studies have shown that ulcerative colitis and CD are associated with increased abdominal adiposity, body weight gain, and colon enlargement. These conditions are also characterized by marked colonic damage and alterations in serum metabolic parameters related to oxidative stress within the colonic mucosa, including excessive ROS production and elevated plasma scavenging activity (PSA). Furthermore, obesity was found to aggravate the severity of acetic acid (AA)-induced ulcerative colitis by enhancing inflammatory responses and increasing the expression of pro-inflammatory cytokines. Notably, SP administration exerted significant protective effects, reducing inflammatory severity and histopathological damage, decreasing lipid peroxidation as indicated by lower MDA levels, and improving antioxidant defenses through enhanced activities of CAT, SOD, and GPX, together with increased levels of non-enzymatic antioxidants such as GSH and SH-G [157]. Furthermore, an oral hydrogel delivery system (SP@Rh-gel) has been developed to facilitate the administration of SP. This strategy was shown to preserve intestinal barrier function, limit the production of pro-inflammatory mediators, and reduce their translocation across the blood–brain barrier into the hippocampus. As a consequence, SP@Rh-gel attenuated neuroinflammatory responses and helped preserve neuroplasticity [161]. In a chronic colitis mouse model, treatment with SP@Rh-gel markedly improved colitis-associated manifestations and significantly reduced anxiety- and depression-like behaviors, highlighting its potential therapeutic value in disorders involving the gut–brain axis. In vitro studies showed that a concentration of SP (50 and 100 μg/mL) alleviated DSS-induced NCM460 cell injury. SP markedly attenuated DSS-induced oxidative stress by limiting excessive ROS production and preserving mitochondrial membrane potential. In vivo, the oral administration of SP at a dose of 300 mg/kg significantly reduced the severity of colonic mucosal injury induced by DSS, while also mitigating inflammation and oxidative damage. These protective effects were associated with enhanced antioxidant defenses, including the increased activities of SOD, CAT, and GSH-Px, as well as the upregulation of colonic tight junction (TJ) proteins, contributing to the maintenance of intestinal barrier integrity [170]. Moreover, orally administered phycocyanin from SP (750 mg/kg) significantly alleviated cognitive impairment in mice by modifying the gene expression profile (*Nfe2l2*, *Prnp*, *Cct4*, *Vegfd (Figf)*, *Map9 (Mtap9)*, *Pik3cg*, *Zfand5*, *Endog*, *and Hbq1a*) associated to AD when altered in the hippocampus of mice [171,172].

#### 6.4.2. Clinical Evidence on Spirulina

In humans, a dose of 500 mg/day SP or a placebo twice a day for 12 weeks in 60 subjects with AD showed a significant improvement in cognitive function by reducing high-sensitivity C-reactive protein (hs-CRP), fasting glucose, insulin, and insulin resistance [173]. Furthermore, SP maxima 70% ethanol extract, three times a day (capsule whole weight 2.4 g/day), has shown the ability to improve memory in the older adults with MCI and the risk of developing AD after 12 weeks [174]. Clinical evidence supporting spirulina is growing. In a randomized, placebo-controlled clinical trial, spirulina supplementation (1 g/day for 8 weeks) significantly improved the total antioxidant capacity and quality of life in patients with ulcerative colitis, suggesting a potential adjuvant role in inflammatory bowel disease [175]. Interest in SP is further supported by a randomized, double-blind, placebo-controlled clinical trial designed to evaluate the effects of supplementation on intestinal permeability, oxidative stress, and symptomatology in patients with irritable bowel syndrome, a major gut–brain axis disorder [176]. Available human evidence includes randomized, double-blind, placebo-controlled clinical trials evaluating the effects of spirulina in inflammatory bowel disease, cognitive decline, and other conditions related to the gut–brain axis and the use of this compound in modulating inflammation, oxidative stress, and neurological health. Table 1 provides an overview of the effects of spirulina on the modulation of the main molecular pathways discussed in this review (↓ inhibition or ↑ activation).

### 6.5. Pterostilbene

#### 6.5.1. Preclinical Evidence on Pterostilbene

Pterostilbene (PTB) is a natural demethylated analogue of resveratrol, which is mainly found in berries, red wine, and *Pterocarpus* species. Its improved lipophilicity and metabolic stability give it a higher bioavailability compared to resveratrol, making it a promising candidate for nutraceuticals. PTB exhibits diverse pharmacological effects, notably antioxidant, anti-inflammatory, anticancer, and neuroprotective properties. Recent studies on PTB in the context of periodontitis are scarce. However, some studies suggest its potential benefits in modulating periodontal inflammation and restoring redox balance [131]. When complexed with cyclodextrin, PTS showed antimicrobial activity against *Fusobacterium nucleatum* and suppressed NF-κB-driven cytokine release in macrophages, while simultaneously upregulating Nrf2-dependent antioxidant genes such as HO-1 and NQO1 [132]. Although no clinical studies in periodontitis are currently available, the mechanistic overlap argues in favor of exploring PTB as an adjuvant in periodontal therapy, possibly through local delivery systems similar to those tested with resveratrol. Indeed, there is solid and recent evidence for the efficacy of PTB in intestinal inflammation, particularly in DSS-induced colitis models. Medicinal chemistry has yielded PTB derivatives with triazole moieties, one of which (compound 8) showed potent inhibition of iNOS and COX-2 expression and significant attenuation of NF-κB and MAPK signaling, resulting in marked protection in acute colitis with an excellent safety profile [133]. Similarly, novel PTB analogues have been identified as specific NLRP3 inflammasome inhibitors. One example is compound D22, which blocked ASC oligomerization and caspase-1 activation, thereby preventing pyroptosis and IL-1β secretion while alleviating DSS-induced colitis in mice [134]. Various innovative delivery platforms have been designed to improve the bioavailability and overcome the pharmacokinetic limitations of native PTB. ROS-responsive nanocarriers not only improved bioavailability in the colon but also enhanced therapeutic benefits by scavenging ROS, modulating dendritic cells, and rebalancing macrophage polarization and T-cell infiltration [135]. Similarly, self-assembled PEGylated PTB micelles improved solubility and stability, suppressed TLR4-mediated activation of NF-κB and MAPK, and promoted epithelial barrier recovery in murine colitis [136]. Furthermore, innovative probiotic–drug conjugates have been engineered to achieve synchronous colonization of adhesive *Escherichia coli* and ROS-responsive release of PTB at colonic lesion sites. This dual delivery system significantly enhanced probiotic survival under harsh gastrointestinal conditions, enabled targeted adhesion to inflamed mucosa, and triggered on-demand release of PTB in response to high ROS levels. In a DSS-induced model of ulcerative colitis, these conjugates exerted synergistic protective effects by reducing the production of the pro-inflammatory cytokines TNF-α, IL-6, and IL-1β, enhancing IL-10 levels, restoring the expression of tight junction proteins, and promoting a healthier gut microbiota profile through the enrichment of beneficial bacterial genera such as *Alloprevotella* and *Lachnospiraceae* [137]. Taken together, these findings suggest that PTB exerts its beneficial effects through multiple molecular mechanisms, including the downregulation of NF-κB/MAPK and TLR4 signaling pathways, as well as the suppression of NLRP3 inflammasome activation, enhancement of tight junction integrity, and modulation of innate and adaptive immunity—advanced formulations are important to achieve site-specific delivery and reduced toxicity. Overall, PTB is proving to be a promising nutraceutical scaffold for IBD. However, translation into clinical use requires standardized formulations, long-term safety evaluation, and pharmacokinetic characterization. Beyond the gut, the neuroprotective properties of PTB have been demonstrated in both cellular and animal models relevant to AD. In amyloid-β1–42-exposed neuronal cells, PTB alone or in combination with diosgenin reduced oxidative stress, inhibited microglial activation, and promoted neuronal survival through the upregulation of Nrf2 and BDNF, suppression of NF-κB signaling, and modulation of autophagy [138]. In APP/PS1 transgenic mice, PTB administration mildly improved cognitive performance, reduced neuroinflammation and amyloid load in the hippocampus, and suppressed TLR4-mediated microglial activation, while suppressing TNF-α, IL-6, and IL-1β expression levels [139]. In addition, PTB was associated with the promotion of autophagy-dependent amyloid clearance, which contributes to the reduction in amyloid burden. The available evidence is consistent with the notion that PTB may attenuate the progression of AD pathology through two mechanisms: enhancement of Nrf2-driven antioxidant defenses and suppression of pro-inflammatory cascades. Although the preclinical evidence for PTB is solid, some limitations must be acknowledged. Most studies are based on chemically induced colitis models or AD transgenic mice, which may not fully recapitulate the human disease. The data on periodontitis are only preliminary as no direct in vivo or clinical studies are yet available, indicating that further research is needed to investigate novel topical delivery systems for periodontal use in humans. In addition, the translation of effective doses from experimental studies to human use is complicated by pharmacokinetic variations, although PTB has better bioavailability compared to resveratrol. Future studies should include well-designed clinical trials to better define the therapeutic potential of PTB in IBD and AD.

#### 6.5.2. Clinical Perspectives on Pterostilbene

In addition to oral and gut disorders, further studies show that PTB activates Nrf2 in various chronic diseases. For instance, in abdominal aortic aneurysm models, PTB activated KEAP1/NRF2, upregulating HO-1/NQO1 and attenuating vascular inflammation [177]. In cyclophosphamide-induced interstitial cystitis, PTB activated Nrf2/HO-1 and suppressed NLRP3 [178]. In skin aging, PTB reduced UVA-induced ROS, restored collagen synthesis, and increased Nrf2/Sirt6 expression [179]. In hyperglycemic endothelial cells, PTB reversed the epigenetic silencing of Nrf2, restoring antioxidant genes and nuclear Nrf2 translocation [180]. While outside oral–gut–brain disorders, these findings confirm Nrf2 activation as a convergent mechanism of PTB. Stilbenes, particularly pterostilbene, have demonstrated promising neuroprotective properties in experimental models of neurodegenerative diseases through the modulation of multiple cellular pathways involved in oxidative stress, neuroinflammation, and mitochondrial function [181]. However, clinical evidence in humans remains limited, highlighting the need for further clinical studies to confirm the therapeutic efficacy of stilbenes in neurodegenerative disorders.

Table 1 provides an overview of the effects of pterostilbene on the modulation of the main molecular pathways discussed in this review (↓ inhibition or ↑ activation).

### 6.6. Icariin

#### 6.6.1. Preclinical Evidence on Icariin

Icariin (ICA) is a flavonoid extracted from *Epimedium* species with potential preventive and pharmacological applications in oral–gut–brain disorders by targeting the Nrf2 pathway and stress resilience genes [182,183,184]. Recent studies demonstrate that under NIR irradiation, ICA + CNF@H exhibited synergistic antibacterial, anti-inflammatory, and antioxidant activities, effectively reducing ROS levels while simultaneously enhancing osteogenic differentiation, thereby representing a promising strategy for periodontitis in vitro and in vivo [185]. Local injection of ICA promoted periodontal tissue regeneration and exerted anti-inflammatory and immunomodulatory function by downregulating IL-1β [140]. Moreover, ICA reversed the inhibitory effect of *P. gingivalis* infection on the periodontal osteogenic differentiation and proliferation of MC3T3-E1 cells in a concentration- and time-dependent manner when administered at doses between 0.1 and 10 μM, with the greatest effect at 10 μM through the downregulation of EphA2-RhoA [141]. In addition, ICA at the concentrations of 20 and 50 µM increased the survival, migration, and osteoblastic differentiation of human periodontal ligament fibroblasts by inhibiting the TLR-4/NF-κB signaling pathway [142]. Finally, zinc-aluminum layered double hydroxide nanosheets (LDHs) incorporating ICA were found to shift macrophage polarization from the M1 to the M2 phenotype, thereby modulating the local immune microenvironment and stimulating the production of cytokines that support oral tissue regeneration [186]. Interestingly, ICA significantly influences the gut microbiota composition and function, thus reducing IBD diseases [187]. Consistent with this, compelling evidence indicates that doses of 80 mg/kg ICA and 150 mg/kg *Panax notoginseng* saponins significantly ameliorate alterations in the abundance, proportion, and function of gut microbiota in AD mouse models [188]. Likewise, a dose of 100 mg of ICA for 15 consecutive days improved the intestinal microbiota integrity by upregulating p-Akt and Nrf2; stress resilience molecules, Sirt 1, Pot1α, BUB1b, FOXO1, Ep300, ANXA3, Calb1, SNAP25, and BDNF; and anti-inflammatory cytokines (IL-10) and by downregulating pro-inflammatory cytokines (IL-1β, TNF-α, IL-2, IL-6, IL-12) and iNOS proteins in old mice associated to age-related disorders [143]. Moreover, treatment with ICA (10 mg/kg/day) attenuated colonic inflammation in a dextran sodium sulfate (DSS)-induced ulcerative colitis mouse model through inhibition of the NF-κB pathway, mediated by changes in p-p65 and p65 expression levels [189]. In particular, the main active metabolites such as icariside II (ICS) and icaritin (ICT) show the ability to slow the worsening of the symptoms of AD via the activation of Nrf2 signaling [190]. Recent evidence suggested that a concentration of 20 mg of ICA in combination with 15 mg/kg/d β-asarone can improve neuronal survival and cognitive function by reducing the production of extracellular amyloid beta peptide (Aβ) in mice [191]. Similarly, Feng and colleagues reported that a dose of 60 mg/kg of ICA significantly reduces intracellular neurofibrillary tangles by inhibiting phosphodiesterase-5 activity and stimulating nitric oxide (NO)/cyclic guanosine monophosphate (cGMP) signaling in the hippocampus and cortex of APP/PS1 transgenic mice [192]. Furthermore, ICA and ICT can inhibit the expression of neuroinflammatory cytokines, such as TNFα, IL-1β, and IL-6, by suppressing high-mobility group protein box 1 (HMGB1)/receptor for advanced glycation end products (RAGE) signaling. Additionally, it can prevent H_2_O_2_-induced neurotoxicity by decreasing ROS production and upregulating Sirt1 [193]. Another piece of evidence suggests that ICA may represent a promising therapeutic candidate for AD due to its ability to modulate the sirtuin 1 (SIRT1) pathway, reduce amyloid-β accumulation, and interfere with key neurodegenerative processes, including neuroinflammation, oxidative stress, autophagy, and neuronal apoptosis [194]. Although the literature on ICA continues to expand and supports its neuroprotective potential through multiple molecular mechanisms, the available evidence is derived predominantly from experimental studies and literature reviews [195].

#### 6.6.2. Clinical Perspectives on Icariin

Clinical evidence remains limited; therefore, clinical trials in humans are needed to confirm the promising results observed in experimental models. Table 1 provides an overview of the effects of icariin on the modulation of the main molecular pathways discussed in this review (↓ inhibition or ↑ activation).

## 7. The Synergistic Role of Probiotics, Polyphenols, and Prebiotics in the Oral–Gut–Brain Axis

### 7.1. Probiotics and Nrf2 Signaling

Emerging evidence highlights that selected probiotics may modulate several molecular pathways by restoring microbial balance, reinforcing epithelial barrier function, and regulating innate and adaptive immunity. Notably, the oral microbiome—a complex ecosystem of bacteria, fungi, viruses, and protozoa—is a significant factor in gut and brain health. Alterations in the composition and function of the oral microbiome have been increasingly associated with the development of systemic inflammatory responses, impairment of BBB integrity, and the amplification of neuroinflammatory processes, all of which are considered important contributors to AD pathogenesis within the framework of the oral–gut–brain axis. Through a complex network of neural, immune, and endocrine pathways, the oral cavity, gastrointestinal tract, and central nervous system engage in continuous bidirectional communication, allowing local microbial disturbances to exert effects at distant sites. Oral pathogens, such as *Porphyromonas gingivalis*, have attracted considerable attention because of their ability to disseminate virulence factors, including lipopolysaccharides (LPS) and gingipains, which can trigger inflammatory responses within the nervous system and contribute to neurodegenerative processes. At the same time, microbiota-derived metabolites, including short-chain fatty acids (SCFAs) and peptidoglycan fragments, may further enhance systemic immune activation and sustain chronic inflammation. Given the central role of microbiome homeostasis in the oral–gut–brain axis, probiotic-based interventions have emerged as promising approaches for preserving cognitive health and potentially lowering the risk of neurodegenerative disorders, particularly AD, through the maintenance of a diverse and balanced microbial ecosystem [196]. Recent evidence has further demonstrated that probiotics can exert antioxidant and anti-inflammatory effects by regulating cellular stress-response mechanisms. More specifically, several probiotic strains have been shown to activate the Nrf2 signaling pathway [197]. However, when this pathway is suppressed or dysregulated, it fails to combat oxidative stress effectively, leading to the accumulation of ROS and the enhancement of pro-inflammatory cytokines, which in turn drive the pathogenesis of many chronic and age-related diseases [198,199,200]. Mechanistically, probiotics act by suppressing NF-κB and MAPK pathways and activating Nrf2 pathway [201]. Notably, several *Lactobacillus* strains, belonging to a major group of lactic acid bacteria, have demonstrated pronounced antioxidant and anti-inflammatory properties in both in vitro and in vivo experimental models [202]. For example, Kullisaar et al. [203] demonstrated the presence of the SOD enzyme in *Lactobacillus fermentum*. Moreover, engineered *Lactobacillus casei BL23* strains producing either CAT or SOD offer promising results in the treatment of Crohn’s disease in studies using mouse models [204].

### 7.2. Probiotics and Periodontal Health

Periodontitis is responsible for the elevated polymorphonuclear leukocytes levels leading to a sustained inflammatory burden and oxidative damage [205]. Nrf2 is crucial in maintaining periodontal tissue homeostasis by regulating stress resilience genes [206]. Accordingly, Vo et al. [207] demonstrated that surfactin isolated from Bacillus subtilis is capable of activating the Nrf2 pathway and enhancing HO-1 expression in human gingival fibroblasts. Moreover, selected probiotics such as *Bifidobacterium animalis* ZK-77 and *Lactobacillus salivarius* ZK-88 alleviate the inflammation caused by pathogenic bacterial species *P. gingivalis* that produce harmful metabolites such as H_2_S and NH_3_ in the oral cavity of rats with periodontitis [208]. Of note, polyphenols alone and in synergy with probiotics such as *Lactobacillus reuteri* have shown a significant therapeutic potential in periodontal disorders [209].

### 7.3. Prebiotics and Clinical Evidence

Prebiotics consist of non-digestible food components, typically including fibers such as inulin, fructooligosaccharides (FOS), and galactooligosaccharides (GOS), that selectively nourish beneficial bacteria in the oral and gut microbiome promoting their growth and metabolic activity. These compounds play a crucial role in the oral–gut–brain axis homeostasis [210]. This active food, has beneficial effects on CNS, and decreases or controls the incidence of AD [211]. Importantly, Zhang et al. demonstrated the therapeutic potential of nicotinamide mononucleotide (NMN)-based synbiotics, in combination with *Lactiplantibacillus plantarum* CGMCC 1.16089 and lactulose, in mitigating AD pathology. Specifically, NMN-based synbiotics were shown to suppress the production of the pro-inflammatory cytokines IL-1β, IL-6, and TNF-α, while also lowering intracellular ROS levels. In addition, this treatment promoted the reduction in Aβ deposition and neuroinflammation and the enhancement of intestinal barrier function by modulating multiple pathological pathways [212]. A prospective randomized double-blind trial enrolled 72 individuals with Parkinson’s disease (PD), who were supplemented with propionic and butyric acids and/or the prebiotic 2′-fucosyllactose for six months in combination with conventional treatment. The study revealed that a dose of 3600 mg of propionic and butyric acid in synergy with the prebiotic treatment significantly improved motor, cognitive, and olfactory function, consistent with systemic effects across multiple brain regions in PD patients [213]. A recent randomized, double-blind, placebo-controlled clinical study indicated that supplementation with probiotics in synergy with prebiotics administered for 90 days significantly restored the composition and metabolic function of the intestinal microbiota compared with the placebo group. Some available human evidence has demonstrated that probiotic, prebiotic, and postbiotic interventions can influence gut microbiota composition and certain cognitive and neurological parameters, highlighting the growing translational interest in microbiome-modulating strategies for disorders of the oral–gut–brain axis [214]. Table 2 provides a schematic summary of probiotic strains and their biological/molecular modulation mechanisms acting on the oral–gut–brain axis as discussed in this review (↓ inhibition or ↑ activation).

## 8. Functional Foods as Autophagy Modulators: Role of Longevity and Healthy Aging Medicine

### 8.1. Autophagy and Cellular Resilience

Autophagy is a highly conserved cellular recycling mechanism that relies on lysosomal degradation to preserve protein quality control, metabolic balance, and cellular adaptation to environmental stressors. Emerging evidence indicates that several functional foods can mimic the biological effects of caloric restriction by activating stress-response and resilience pathways, thereby stimulating autophagic processes and potentially contributing to healthy aging and increased longevity [215]. By recycling damaged organelles, misfolded proteins, and toxic aggregates into basic metabolic precursors (e.g., amino acids, fatty acids), it promotes cell survival, prevents the accumulation of cellular debris, and directly contributes to longevity and healthy aging. The progressive decline of autophagic efficiency is considered a characteristic feature of biological aging and contributes to the pathogenesis of numerous age-related disorders, such as neurodegenerative and metabolic diseases. Bioactive natural compounds derived from plants and foods, like polyphenols, flavonoids, alkaloids, terpenoids, and probiotics, have been identified as important regulators of autophagic processes. Through their ability to enhance cellular stress resistance and homeostatic mechanisms, these compounds are increasingly considered promising nutritional tools for supporting healthy aging and longevity [216]. Their effects are mediated by the modulation of several signaling networks involved in autophagy regulation, including Nrf2, AMPK, PI3K/AKT/mTOR, SIRT1, and FOXO pathways. In addition, these bioactive compounds contribute to the attenuation of oxidative stress, chronic inflammation, and mitochondrial dysfunction, all of which are closely linked to aging and age-related diseases [217]. Principally, they act as a survival mechanism, clearing damaged cellular components by activating AMPK (energy sensor) and inhibiting mTORC1 (nutrient sensor) to maintain cellular homeostasis [218]. For instance, resveratrol activates the Nrf2 pathway and related cellular resilience proteins such as SIRT1 to regulate the autophagy process in the brain [219]. Among the members of the sirtuin family, SIRT1 plays a central role in the regulation of lifespan and cellular homeostasis. Its activity promotes autophagy through the deacetylation of several autophagy-related proteins, including ATG5, ATG7, and LC3, thereby facilitating autophagosome biogenesis and maturation [220]. In addition, SIRT1 influences the activity of transcriptional regulators such as FOXO and PGC-1α, which are involved in the coordinated control of autophagy, mitochondrial biogenesis, and antioxidant responses mediated by Nrf2 [221]. Since SIRT1 activity depends on the availability of nicotinamide adenine dinucleotide (NAD^+^), the age-associated reduction in NAD^+^ levels may compromise these protective pathways, leading to impaired autophagic function and accelerating cellular aging. The age-related decline in nicotinamide adenine dinucleotide (NAD^+^) levels impairs SIRT1 activity and subsequently autophagy, accelerating cellular aging [222].

### 8.2. Bioactive Compounds Targeting Autophagy

Importantly, PTB supplementation promoted anti-inflammatory immune phenotypes and cellular homeostasis by activating autophagy. Specifically, it downregulated the *Slc7a5* gene that regulates mTOR activity through the increased uptake of essential amino acids and stress response *Rtp4* gene for enhancing healthy longevity [223]. In Caco-2 cells, indicaxanthin, a bioactive pigment derived from *Opuntia ficus-indica*, has been shown to stimulate autophagic activity by increasing the expression of the autophagy-related markers LC3-II and Beclin1, while also promoting the formation of autophagolysosomes [224]. Similarly, another study found that ICA effectively improved neuronal degeneration associated with aging by upregulating autophagy-related proteins LC3B, Beclin1, and p-AMPK and downregulating the expression of p62, p-mTOR, and p-ULK1 in the cortex and hippocampus of aging rats [225]. Kaempferol has been shown to stimulate autophagic activity in human endothelial cells, while gallic acid exerts similar effects in both CCD-18Co cells and animal models. These effects appear to be mediated through the suppression of the PI3K/AKT/mTOR signaling pathway, a key regulator of autophagy [226,227]. A recent in vitro study demonstrates that synergistic treatment with resveratrol and lithium chloride reduces oxidative stress and enhances antioxidant and autophagic pathways via the activation of the p62/Keap/Nrf2 pathway [228].

### 8.3. Probiotics and Autophagy

Probiotic supplementation has been associated with increased beneficial microbial populations and enhanced production of SCFAs, particularly butyrate, produced through the fermentation of prebiotic substrates. These effects contribute to the modulation of intestinal homeostasis by reducing the production of pro-inflammatory cytokines, attenuating age-related cellular alterations, including mitochondrial dysfunction, and improving autophagic activity. Collectively, these mechanisms have been linked to improvements in gastrointestinal and cognitive function in experimental models of AD [229]. Further evidence has highlighted the ability of selected probiotics, including Milmed yeast, to exert antioxidant and anti-inflammatory effects through the restoration of autophagic pathways. In particular, studies conducted in BV-2 microglial cells and in the in vivo model organism *C. elegans* demonstrated that Milmed supplementation promotes autophagic processes while simultaneously reducing oxidative stress and inflammatory responses [230]. Treatment with either Milmed YPD-cultured yeast or its derived dried formulation enhanced autophagic flux by upregulating the expression of key autophagy-related genes, including Beclin-1, ATG7, LC3, and p62, while suppressing mTOR expression. In parallel, Milmed strengthened cellular antioxidant defenses by increasing the expression of Nrf2 and its downstream antioxidant enzymes SOD1 and GPX in BV-2 microglial cells. The findings obtained in *C. elegans* corroborated these observations, confirming a significant reduction in reactive oxygen species (ROS) levels together with enhanced stress resistance and prolonged lifespan, thereby supporting the role of autophagy restoration in promoting cellular resilience and healthy aging [230]. Finally, emerging evidence suggests that interventions based on combinations of micronutrients, probiotics, collagen peptides, cannabidiol, and targeted dietary and lifestyle modifications may further support autophagic activity. Through the coordinated modulation of cellular stress-response pathways, these approaches have been proposed as potential strategies to counteract age-related decline and slow the progression of AD, although additional translational and clinical studies are needed to validate these findings in humans [231]. However, since most of the available evidence is derived from preclinical studies, further clinical investigations are needed to confirm the potential of precision nutrition to modulate autophagy and promote healthy aging in humans. Collectively, autophagy dysregulation leads to accumulation of protein aggregates, dysfunctional mitochondria, chronic inflammation, and genomic instability, all of which contribute to aging and limit lifespan. Precision nutritional interventions targeting critical pathways of cellular resilience response and molecular checkpoints are needed to identify responders, define timing, and optimize dosing regimens, ultimately promoting balanced autophagic activity and healthy aging, resulting in prolonged longevity in humans.

## 9. Advances in Precision Nutritional Medicine: A Multi-Omics Approach in the Oral–Gut–Brain Axis

Precision nutrition is an emerging concept that shifts dietary guidance from population-wide averages to highly individualized nutritional recommendations. It leverages multi-omics, i.e., genomics, metabolomics, oral and gut microbiome profiling, and environmental exposure, to identify exactly how genetic variations modulate responses to specific nutrients and dietary patterns. Importantly, it translates a person’s unique molecular and biochemical footprint into actionable, targeted nutritional strategies to reduce inflammation, neutralize oxidative stress and maintain oral–gut–brain homeostasis at cellular and systemic levels [232,233]. Essentially, precision nutrition is built on the concept that a “one-size-fits-all” diet is inadequate. Because every human body absorbs, synthesizes, and reacts to nutrients differently, this framework utilizes advanced data to optimize individual health [234].

### 9.1. NFE2L2 and KEAP1 Genetic Variants Modulated by Functional Foods

Epigenetic modifications, such as DNA methylation, histone modifications, and interactions with non-coding RNAs are essential for regulating Nrf2 expression by modulating chromatin architecture and gene accessibility [235]. The nuclear factor, erythroid 2 like 2 *(NFE2L2)* gene, encodes the Nrf2 protein, the master regulator of antioxidant and cellular stress defenses. It is kept in check by the KEAP1 protein. In normal conditions, *NFE2L2* and *KEAP1* genes regulate cellular resilience responses, determining how our body processes dietary interventions, while the gut microbiome mediates 20–40% of metabolic responsiveness by breaking down specific nutrients. Genetic variations in the *NFE2L2* gene can alter how effectively our body produces this protein, sometimes leaving individuals more susceptible to chronic inflammatory or metabolic conditions [236]. Of note, single nucleotide polymorphisms (SNPs) and haplotypes in the *NFE2L2* and *KEAP1* genes influence baseline antioxidant capacity, disease risk, and how effectively the body utilizes functional foods [237]. Moreover, genetic variations within DNA binding domains can alter responsiveness to microbial and dietary signals, meaning individuals with different genotypes may require varying concentrations of phytochemicals to achieve the same antioxidant effect [238]. Functional foods documented to modulate Nrf2 signaling act by reversing hypermethylated states in the CpG islands of *NFE2L2*, via the inhibition of DNA methyltransferases (DNMTs) and histone deacetylases (HDACs), through the induction of ten-eleven translocation (TET) enzymes, or by inducing miRNA to target the 3′-UTR of the corresponding mRNA transcripts in oral–gut–brain axis disorders. In this new perspective, precision nutrition targeting the Nrf2 pathway and detoxifying enzymes can enhance IEB and BBB resilience. Sulforaphane, a functional food, promotes targeted demethylation of the *Nrf2* promoter, confirming the plausibility of diet-linked epigenetic regulation of antioxidant pathways [239]. In addition, sulforaphane upregulated Nrf2 expression and promoted Nrf2 nuclear translocation via decreasing the DNA methylation levels of the Nrf2 promoter, leading to antioxidant and anti-inflammatory effects in a cellular model of AD [240]. Moreover, PTB-linked epigenetic regulation reverses gene silencing, boosting Nrf2 expression to enhance cellular resilience response against oxidative stress and inflammation [180]. Specifically, PTB reverses epigenetic alterations by demethylating the CpG island of the Nrf2 promoter region. The reactivation of Nrf2 triggers the transcriptional activation of stress resilience response genes, including SOD2, which combats ROS-mediated stress in many chronic diseases. Furthermore, sodium butyrate dose-dependently decreased *NRF2* gene expression through regulating the *KEAP1* promoter methylation, which further regulates *NRF2*-target genes and contributes to colon cancer prevention and therapy [241]. Collectively, we hypothesize that the diagnostic use of nutritional biomarkers of cellular resilience response, specifically the Nrf2-ARE pathway, which regulates genes for glutathione synthesis and phase II detoxification, reflect how well a body adapts to chronic environmental stressors and xenobiotics (e.g., MNPs) as an intrinsic response to the ingestion of specific nutrients. By measuring these genetic and enzymatic indicators in biological fluids, clinicians can objectively assess the systemic nutrient status in the presence of various systemic environmental stressors or pollutions [242]. These indicators are highly effective for diagnosing subclinical deficiencies by assessing systemic adaptations rather than relying solely on traditional markers [15]. Unlike traditional approaches based on average nutrient intake rates, the use of precision nutrition combined with biomarkers of cellular resilience could identify specific oral and gut disorders as well as nutrient deficiencies associated with an increased risk of AD. This opens up opportunities for targeted and safe correction, which is especially important in the context of pathogenetic mechanisms associated with oxidative stress, neuroinflammation, and compromised physiological barriers (oral, intestinal, and intracranial) induced by MNPs [243].

### 9.2. Oral and Gut Microbiome-Related Differences in Response to Precision Nutritional Interventions

The complex interplay between diet, the gut microbiome, and human physiology, three deeply interdependent systems, underlies the substantial inter-individual variability observed in metabolic and clinical responses to precision nutritional interventions [244]. Microbiome-based precision dietary interventions use information about an individual’s gut bacteria to guide nutrition and improve metabolic health [245]. Research demonstrates that identical foods cause wildly different metabolic responses depending on a person’s microbiome. Indeed, disruption of microbiota composition or function (dysbiosis) has been implicated in the pathogenesis and progression of numerous metabolic, inflammatory, and even SNC disorders. For instance, in individuals with periodontitis, it enriches microbial pro-inflammatory pathways and depletes SCFAs and vitamins in both saliva and stool [246]. Interestingly, probiotics acts as a potential adjunctive therapy in periodontitis management [247]. In particular, probiotic *Lactobacillus* promoted the disappearance of swelling of the peri-implant mucosa, with reduced bleeding on probing and good control of plaque and pigmentation [248]. Recent human studies revealed that subgingival application of *Lactobacillus reuteri* probiotic improved the clinical and microbiological parameters of participants, reducing *P. gingivalis* levels compared to the untreated group in peri-implantitis patients [249,250]. In addition, in vitro experiments of the anti-inflammatory capacity of six isolated probiotic strains and their combinations revealed that *Lacticaseibacillus rhamnosus*, *Weissella confuse*, and especially their combination exhibited superior anti-inflammatory activity compared to other strains against periodontal pathogens [251]. Functional dietary fiber- or prebiotics-based Mediterranean patterns have been shown to positively modulate gut microbiota composition, promote beneficial bacteria (*Bifidobacterium*, *Faecalibacterium prausnitzii*, and *Roseburi*), and enhance SCFA production such as acetate, propionate, and butyrate, thereby reinforcing intestinal barrier and reducing inflammation [252]. Despite these advances, translating precision nutrition into clinical practice faces persistent challenges, including inter-individual variability in dietary responses and the need for large-scale, long-term validation studies.

### 9.3. Metabolomics-Based Nutritional Approaches

Metabolomic profiling directly evaluates the complex biochemical interactions between diet, host physiology, and oral and gut microorganisms. By identifying and measuring these microbial-derived metabolites, researchers are uncovering clinical biomarkers to design data-driven and precision nutritional therapies [253]. Interestingly, periodontal staging was marginally associated with some salivary metabolites; other factors such as systemic antibiotic use may have a much more profound effect on the microbial metabolites in saliva [254]. In the context of precision nutrition, garlic-derived exosome-like nanovesicles (GaELNs) demonstrated potent anti-inflammatory, antioxidative, and metabolism-enhancing properties, offering significant therapeutic potential for the treatment of periodontitis by increasing the Nrf2 pathway and the PHGDH/PI3K/AKT pathway [255]. The specific multi-omics study exploring the oral–gut axis in early pregnancy is highly relevant for establishing the importance of pre-conceptional and first-trimester oral health [256]. Specifically, the study analyzed subgingival plaque, saliva, serum, and fecal samples from 54 pregnant women in their first trimester, comparing 31 subjects with maternal periodontitis to 23 healthy controls. Fecal analysis identified the bacterial genus Coprococcus as a distinguishing biomarker in women with periodontitis, linking it to subgingival periodontal pathogens. Subjects with periodontitis showed significantly elevated levels of specific fecal metabolites, primarily L-urobilin and kynurenic acid [256]. A prospective cohort study has shown that the increased risk of Crohn’s disease associated with ultra-processed food may be driven by a relative deficiency of protective metabolites such as docosahexaenoic acid (DHA) [257]. The analysis of the hippocampus, midbrain, temporal and entorhinal cortex, and their respective analysis of brain-derived EVs indicated that the metabolic profiles of different brain areas were distinct and showed some correlation between the metabolome of the tissue [258]. The pathway enrichment analysis of the common metabolites showed that the alanine, aspartate, and glutamate pathway and the arginine, phenylalanine, and tyrosine pathway were the most significant ones in the separation between AD patients and controls [258]. Overall, metabolomics serves as a fundamental bridge in nutritional science, revealing how diet and microbial activity dynamically shape host biology. By monitoring small-molecule metabolites and assessing each individual’s unique metabolomic profile and metabolic response to functional nutrients, it will be possible to discover novel therapeutic targets and clinical biomarkers to personalize and refine nutrition, particularly by addressing the complex bidirectional crosstalk along the oral–gut–brain axis.

### 9.4. Precision Nutrition to Translate from Preclinical Data into Clinical Applications in Human Populations Exposed to MNPs

Precision nutrition aims to mitigate cellular damage, oxidative stress, and inflammation caused by MNPs. Since MNPs are ubiquitous in food, water, and air, nutritional interventions focus on limiting intestinal absorption, protecting intestinal barrier integrity, and enhancing Nrf2 resilience signaling through personalized polyphenol-rich dietary patterns [259]. Currently, precision clinics offer specific advanced panels like NANOXPACE bioscience testing to evaluate systemic inflammation and MNP accumulation through stool and blood analyses to personalize interventions, i.e., translating clinical data into targeted therapies and lifestyle adjustments to reduce body burden [260]. This panel monitoring allows a deeper understanding of the long-term health effects associated with the presence of MNPs within tissues and bodily fluids [260]. Integrating specific probiotics in synergy with nutritional polyphenols creates a symbiotic strategy that mitigate MNPs’ toxicity. This synergistic action fortifies the oral and intestinal mucosal barriers, inhibits inflammatory signaling cascades, and upregulates cellular antioxidant defenses along the gut–brain axis to prevent systemic neurotoxicity [261,262]. Specific probiotic strains, such as *Lactiplantibacillus plantarum* and *Clostridium dalinum*, upregulate tight-junction proteins (e.g., ZO-1, occludin), which restricts the translocation of MNPs and endotoxins into the bloodstream [261]. A preliminary study of environmental MNP exposure in human biological sampling (urine) was conducted on six volunteers from different cities in the south of Italy and suggests that MPs (4–15 μm size) of irregular shape could pass through the gastrointestinal tract and are eliminated through biological processes [263]. Also, Calikanzaros and coworkers in a recent cross-sectional, population-based study of 50 healthy adults in Barcelona quantified mass concentrations of MNPs in stool, urine, tap water, and food samples, estimating the average daily intake through diet, drinking water, and lifestyle [264]. The detection of MNPs in urine suggests systemic circulation, particularly for smaller particles capable of entering the bloodstream, being filtered by the kidneys and subsequently excreted. Using HPLC-MS analysis, particles as small as 0.7 µm were quantified, suggesting that diet, including food packaging, is a major source of oral exposure to MNPs [264]. Despite recent advances, studies investigating clinical nutrition and MNP-induced systemic toxicity in humans remain limited; therefore, a multidisciplinary approach is urgently needed to fill these gaps. We believe that, by integrating clinical nutritional medicine, molecular biology, environmental toxicology, neuroscience, and engineering with advanced methodologies and analytical protocols, it will be possible to implement innovative precision interventions to monitor circulating MNP concentrations and their potential transport routes in biological fluids and tissues. This interdisciplinary approach, combined with precision and personalized clinical nutrition targeting cellular resilience mechanisms, can prevent MNP-induced damage to cells, tissues, and organs, and it can ultimately guide diagnosis, prognosis, and future therapies in oral–gut–brain axis disorders.

## 10. Artificial Intelligence in Precision Nutritional Medicine

The revolutionary approach with artificial intelligence (AI) in the field of nutrition represents a paradigm shift in the research and development of innovative therapeutic strategies from bench to clinic also known as “translational medicine”. The application of machine learning algorithms to the study of the gut microbiome and brain function can enhance the multi-omics analysis of complex biological interactions, facilitating the identification of hidden neuronal pathways, precise prediction of diseases, and the personalization of therapies and diets based on individual microbiotic and neuronal profiles as well as the discovery of novel potential biomarkers contributing significantly to the holistic well-being of individuals. Furthermore, AI-based approaches enable in-depth exploration of the preventive and therapeutic effects of the nutraceuticals, particularly the synergy between polyphenols and/or probiotics, to improve their delivery and efficacy in the organism during gut dysbiosis and neuroinflammation leading to the onset of neurodegenerative disorders. Nutritional research through the use of advanced algorithms could serve as an intrinsic indicator for discovering intestinal and neuronal markers associated to optimal intestinal and brain health or the presence of specific disorders. In this new perspective, the AI approach allows a faster and more efficient optimization of polyphenols and probiotics, overcoming obstacles such as variability among individuals and adverse conditions of the upper gastrointestinal tract [265,266,267]. Notably, bioinformatic tools like probiotics, which apply advanced machine learning techniques, are transforming the way therapies related to host gastrointestinal survival/establishment, carbohydrate utilization, drug resistance, and virulence factors are discovered and optimized [265]. These technologies enable high-throughput screening, predictive modeling of gut microbiota, and the customization of treatments, shifting the focus from “one-size-fits-all” to personalized, data-driven precision medicine. Investigating the metabolomic profile of the gut microbiota is essential for clarifying its influence on both physical and mental health, although the complexity and dynamic nature of microbial ecosystems have traditionally limited such analyses. Recent advances, including the development of in vitro systems that mimic the intestinal microbial environment, such as ABIOME, together with the application of predictive computational approaches like multivariate adaptive regression splines (MARS), have improved the ability to model microbial metabolism. These technologies enable the prediction of the metabolic output of specific probiotic formulations and facilitate the assessment of their potential therapeutic applications [267]. AI techniques, such as machine learning tools, are increasingly being used in precision diagnosis, with the ultimate goal of providing a comprehensive overview of how AI and the nutritional approach are integrated into modern medicine.

### 10.1. AI-Driven Strategies for Alzheimer’s Disease

**Figure 3:**
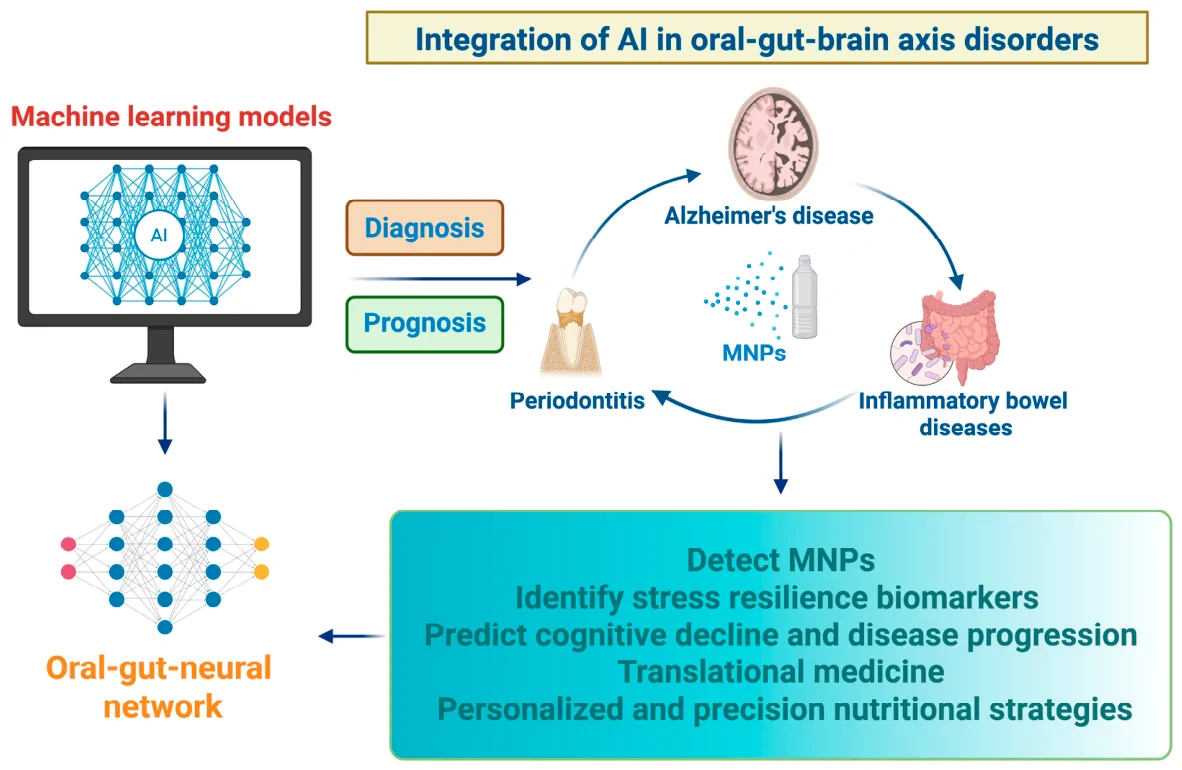
Application of AI in oral–gut–brain axis disorders. Created in BioRender. https://BioRender.com/21iwwfs (accessed on 20 July 2026). The arrows indicate the correlation between machine learning models, the early identification of Alzheimer’s disease, and biomarker detection.

This study protocol aims to develop a personalized dietary supplement for individuals with AD by integrating clinical, dietary, and gut microbiota data through AI and network-based analytical approaches. In addition, a pilot investigation will evaluate the short-term impact of this intervention on microbiota composition, microbial functionality, and plasma metabolomic profiles to identify potential modifiable biomarkers. The integration of AI-driven tools with microbiome research may facilitate the implementation of precision nutrition strategies in AD, with the potential to modulate disease-associated microbial signatures and systemic biomarkers, thereby supporting the development of innovative therapeutic approaches and advancing personalized care in neurodegenerative disorders [268]. A recent study developed an ultrasensitive point-of-care biosensing system based on a polyphenol red (pPhR) platform coupled with highly porous gold (HPG) electrodes for the detection of phosphorylated tau 181 (p-tau181) in undiluted human plasma and serum samples. This approach demonstrated considerable potential for facilitating the early identification of AD in both primary healthcare settings and home-based screening applications. Owing to its adaptable architecture, the platform may also be configured to detect additional neurodegeneration-related biomarkers, including Aβ40/42, (NfL) and p-tau217, thereby supporting the future development of multiplex diagnostic devices for more comprehensive neurological assessment [269] (Figure 3). Overall, there are currently few studies on the use of AI in oral–gut–brain axis disorders, including its involvement in quantitative feature extraction from machine learning, deep learning, and computer-aided diagnosis approaches, as well as the effect of AI on improving multi-omics analysis and the efficacy of nutritional-AI based approaches in the prevention and management of patients with oral, gut, and neurological disorders aiming to identify novel biomarkers of stress resilience and personalize nutritional and precision therapeutic strategies.

### 10.2. AI to Detect AD Biomarkers and Predict Cognitive Decline

Recent research into high-dimensional proteomics data for AD diagnostics suggests that traditional batch effect correction methods have a minimal impact on model performance compared to robust feature selection. The machine learning process for identifying key protein candidates including fatty acid-binding protein 3 (FABP3) and glutamate oxaloacetate transaminase 1 (GOT1) proved more critical for model stability in detecting AD pathology than extensively correcting for batch effects. The findings show the potential for translating biomarkers from related neurodegenerative conditions, such as idiopathic normal pressure hydrocephalus (iNPH), into AD diagnosis in both ventricular and lumbar CSF samples [270]. Recent advances in machine learning applications have enabled the development of multiclass predictive models aimed at identifying molecular signatures associated with the transition from MCI to AD [271]. Using this approach, researchers identified several metabolites and proteins with potential prognostic value. Experimental analyses demonstrated that microglial cells release oleamide through extracellular vesicles, while preliminary human data revealed elevated plasma oleamide levels in AD patients compared with cognitively healthy individuals. The study further highlighted phospholipase A2 (PLA2G1B), properdin (CFP), alpha-synuclein (SNCA), and junctophilin-3 (JPH3) as relevant biomarkers associated with disease progression, with JPH3 emerging as a particularly promising candidate due to its role in neuronal function. In addition, post hoc analyses from the EXPEDITION3 trial indicated that machine learning algorithms could support the optimization of AD clinical trial design by identifying participants at greater risk of cognitive deterioration, thereby facilitating more efficient evaluation of anti-amyloid therapeutic strategies [271]. Based on a post hoc analysis of the EXPEDITION3 study, predictive models using machine learning can improve the design of AD clinical trials by identifying participants likely to experience cognitive decline in order to optimize anti-amyloid treatments [272]. Based on the study performed by Chen et al., the integration of MemTrax and blood-based biomarkers (BBMs) using machine learning models, specifically in a cohort of 349 participants, showed a significant improvement in the ability to differentiate AD amyloid status and biomarkers, especially for p-Tau181/Aβ42 in predicting early AD diagnosis, potential reduction in invasive tests, and improving patient management in primary care settings [273]. Using three machine learning approaches, namely, Least Absolute Shrinkage and Selection Operator (LASSO), Support Vector Machine Recursive Feature Elimination (SVM-RFE), and Random Forest, researchers narrowed these down to six core diagnostic genes—SCG3, CD86, VGF, PRKCG, SPP1, and TPI1—associated with AD. Particularly, the authors suggested that SCG3 is involved in secretory granule biogenesis and neurotransmitter storage, and it is a potential, reliable early diagnostic biomarker for AD [274].

### 10.3. AI to Detect MNPs in the Oral–Gut Microbiome Axis

Rezvani et al. investigated 614 and 3924 plastic particles generated from PE and PET, respectively, using FastSAM, an artificial intelligence-assisted automated annotation tool, to characterize MNP particle abundance, size distribution, and morphological features. By combining realistic environmental weathering simulations with AI-based image analysis, the study provided a comprehensive assessment of the relative contribution of PE and PET materials to plastic pollution. The results showed that PET was more prone to fragmentation than PE, generating a substantially greater proportion of nanoparticles, which accounted for 57.6% of the particles produced from PET compared with 24.9% for PE, suggesting that PET may represent a particularly important source of environmentally relevant MNPs [275].

### 10.4. AI and Molecular Mechanisms of MNPs Inducing Periodontitis

Recent experimental studies employing gingival fibroblasts (GFs) exposed to PET-MPs have demonstrated dose-dependent cytotoxic effects, accompanied by increased oxidative stress and activation of pro-inflammatory responses. Specifically, PET-MP exposure significantly increased the expression of several inflammation-associated markers, including caspase-3, KDR, PIM2, PTGS2, MTOR, and MAPK14, while reducing the expression of AKT1 and ALPL. Collectively, these findings provide mechanistic evidence supporting a link between PET-MP exposure and the progression of periodontitis through the interplay of oxidative stress and inflammatory pathways, offering new insights into the potential contribution of MNPs to periodontal disease pathogenesis [95].

### 10.5. AI and Molecular Mechanisms of MNPs Inducing Ulcerative Colitis

Animal studies demonstrated that exposure to PET-MPs in combination with DSS significantly increased the expression of CTSK, PDE4B, and PFKFB3, while markedly decreasing NAAA expression compared with DSS treatment alone. These findings provide important insights into the molecular pathways through which PET-MPs may contribute to the development and exacerbation of ulcerative colitis, thereby improving our understanding of the potential health risks associated with PET-MP exposure [276].

## 11. AI and Clinical Translation in the Oral–Gut–Brain Axis

AI accelerates clinical translation in the oral–gut–brain axis by decoding complex, bidirectional interactions between the oral and gut microbiomes with neuroimaging data into predictive, actionable clinical tools [277]. Traditional statistical methods rely on linear assumptions and fixed distributions, making them inadequate for the sparsity, non-linearity, and compositional constraints (relative abundances) of oral and gut microbiome data [278]. AI overcomes these limitations by utilizing frameworks that map ecological coexistence and molecular host-immune interfaces, microbiome composition profiles, whole-brain functional connectivity matrices, and multi-omics. By understanding the oral–gut–brain axis, clinicians can better stratify patients based on their specific microbial and neuroimaging profiles, paving the way for targeted dietary or probiotic interventions aimed at preserving cognitive function [279].

### 11.1. AI and Clinical Translation in Periodontitis

A recent longitudinal study revealed that AI models can predict periodontitis progression, supporting early detection strategies by combining clinical data with salivary biomarkers such as IL-1β, ultimately improving predictive accuracy for precision and personalized interventions [278]. Finally, another important study demonstrated that neutrophil extracellular trap-related genes (NRGs) hold significant promise as potential diagnostic parameter for periodontitis. Utilizing machine learning algorithms accurately evaluated disease severity and tissue destruction. Notably, both immune-inflammatory pathways CXCR4 and HIF1A were validated as key upregulated diagnostic biomarkers, highlighting crucial targets for future periodontal therapies [280].

### 11.2. AI and Clinical Translation in IBD

AI is transitioning from a research concept to a clinical reality in IBD [281]. An observational study identified and validated a predictive model (IBD-RESPONSE) to correlate gut microbiome and treatment response in IBD [282]. A multi-omics AI model predicted enteral nutrition response in pediatric IBD patients, supporting their potential use in precision nutrition and personalized care strategies [283]. Specifically, this novel tool has predicted treatment response to serum metabolites (2-hydroxyglutaric acid), fecal metabolites (3-methyladipic acid), and microbial taxa (family Bifidobacteriaceae and genus CAG-56) in a cohort of 50 children [283]. Moreover, transformer-based foundation models such as BiomeGPT have revolutionized microbiome analysis by parsing quantitative species-level abundances to identify disease-specific signatures rather than generic dysbiosis [284]. Specifically, this database accurately distinguishes taxonomic profiles to differentiate health states from dysbiosis in conditions such as inflammatory bowel disease, paving the way for targeted therapeutic interventions, biomarker discovery, disease stratification, and microbiome-driven precision medicine [284]. By targeting microbial dysbiosis, these strategies reduce systemic inflammation associated with periodontitis and IBD, helping to mitigate the neuroinflammatory cascades linked to AD. AI platforms compute highly specific synbiotic combinations (probiotics + prebiotics) and dietary adjustments designed to increase beneficial bacteria (e.g., *Faecalibacterium prausnitzii*, *Akkermansia muciniphila*) while starving pro-inflammatory pathobionts [285]. Therefore, AI integrates comprehensive multi-omics data including blood inflammatory markers (like hs-CRP) and salivary/fecal metagenomes to generate individualized and precision nutritional strategies and synbiotic plans [286].

### 11.3. AI and Clinical Translation in AD

AI models analyze coordinated changes between gut microbes and brain networks (often using fMRI scans) to identify reliable biomarkers in AD [287]. Xu et al. validated a network topology-based deep learning framework known as NETTAG that integrates multi-omics data to identify putative risk genes in AD [288]. This network identified 156 AD risk genes enriched in drug targets. Combining network-based prediction and retrospective case-control observations with 10 million individuals, the authors identified that the usage of four drugs (ibuprofen, gemfibrozil, cholecalciferol, and ceftriaxone) is associated with reduced likelihood of AD incidence [288]. Overall, clinical translation of medical AI and diagnostic models requires overcoming the “proof-of-concept” barrier, larger multicenter datasets, harmonized preprocessing pipelines, external validation, calibration reporting, and evaluation against clinically realistic comparators and decision points.

## 12. Challenges and Limitations of the Study

The current knowledge regarding the overall effects of MNPs on the human body is still fragmented and poorly integrated across different scientific fields. The main challenges arise from the methodological complexity of detecting and analyzing such small particles and accurately describing their characteristics, as well as the lack of shared techniques for identifying them in organs and tissues. In addition, the lack of standardized contamination control criteria makes it difficult to compare the results obtained from different studies and limits the reliable assessment of unaware human exposure, also due to the great variability of MNPs. A further obstacle is the lack of experimental mouse models to study dose-related effects, which are essential for understanding the hazards and biological mechanisms associated with particles with different properties and sizes. Furthermore, long-term studies in humans are still insufficient to clarify the chronic consequences of prolonged exposure. Currently, there is growing interest in the potential link between MNPs and the balance of the oral–gut–brain axis. Interference with the oral and intestinal microbiota represents a still largely unexplored field of research, yet it might potentially be relevant in understanding various functional disorders. Despite a rapidly growing body of evidence, several gaps remain. Clinical studies in periodontitis and IBD are encouraging but limited in sample size and duration, while neuroprotective findings are restricted to animal models. Moreover, variability in spirulina formulations (powder, peptides, and nanoformulations) complicates cross-study comparisons and translation to clinical practice. Future research should focus on standardized extracts, pharmacokinetic assessments, and well-powered randomized trials to establish robust clinical guidelines. Additionally, mechanistic investigations are warranted to dissect the contribution of specific bioactive compounds (e.g., phycocyanin vs. peptides) targeting Nrf2 activation and immunomodulatory molecules, including pro-inflammatory and anti-inflammatory cytokines. Addressing these challenges requires fostering integration between diverse disciplines, such as biology, medicine, nutrition, and neuroscience, and promoting greater sharing of data and knowledge. Polyphenols are compounds that may contribute to the modulation of the oral–gut–brain axis; however, a major limitation is their reduced absorption. Their poor solubility and rapid metabolic transformation significantly reduce their biological effectiveness. The use of advanced solutions, such as advanced nanoformulation strategies, can improve the stability and availability of these molecules, promoting their absorption. These approaches could help support the balance between the microbiota and brain function, even in the presence of critical environmental factors such as MNPs.

## 13. Available Human Evidence and Translational Perspectives

Despite the growing interest in the use of bioactive compounds such as artichoke (*Cynara scolymus*), *Opuntia ficus-indica*, spirulina, pterostilbene, icariin, and quinic acid for disorders involving the oral–gut–brain axis, the available clinical evidence remains limited. Most published studies consist of cellular models and preclinical investigations. Regarding periodontitis, some evidence suggests that compounds with antioxidant and anti-inflammatory properties may contribute to the modulation of local inflammation and oral microbiota homeostasis; however, clinical studies specifically investigating the compounds discussed in the present review are still scarce and generally characterized by small sample sizes. Similarly, in the context of IBDs, several bioactive compounds have shown promising effects on inflammatory markers and gut microbiota composition, but evidence from controlled clinical trials remains insufficient to support definitive clinical recommendations. For AD and neurodegenerative disorders, the currently available evidence is derived almost exclusively from experimental studies. Although several compounds have demonstrated neuroprotective properties through the modulation of Nrf2 signaling, oxidative stress, and neuroinflammation, clinical studies confirming their efficacy in humans are still lacking. Finally, a particularly relevant aspect concerns exposure to MNPs.

To date, no clinical studies have directly evaluated the ability of these compounds to prevent or mitigate the biological effects associated with MNP exposure in humans. Therefore, the hypothesis of a protective role is mainly based on the extrapolation of shared biological mechanisms, including the modulation of oxidative stress, inflammation, and biological barrier integrity. Importantly, most of the available evidence supporting the protective effects of these bioactive compounds derives from studies investigating oxidative stress, inflammation, gut dysbiosis, and barrier dysfunction in experimental models that are not directly related to MNP exposure. Therefore, many of the proposed protective effects should be considered indirect and based on biologically plausible mechanisms shared across different pathological conditions rather than on direct evidence demonstrating the prevention or reversal of MNP-induced toxicity. While these findings suggest the probable involvement of functional foods, their specific efficacy against MNP-associated damage remains to be established through experimental and clinical studies.

Notably, the available human evidence includes two randomized, double-blind, placebo-controlled clinical trials and one randomized clinical trial protocol evaluating spirulina supplementation in inflammatory bowel diseases, cognitive decline, and gut–brain axis-related disorders, suggesting a growing translational interest in its therapeutic potential. Likewise, preliminary human studies on Opuntia support its potential role in modulating gut health, oxidative stress, and gut–brain axis functions. Similarly, clinical evidence for *Cynara scolymus* indicates that artichoke-derived inulin favorably modulates gut microbiota composition by increasing beneficial bacterial populations, supporting its involvement in gut–brain axis regulation through prebiotic mechanisms. In contrast, human evidence for icariin, pterostilbene, and quinic acid remains extremely limited, and clinical studies specifically evaluating their effects on oral–gut–brain axis disorders are currently lacking. Overall, the limited number of available clinical studies and the absence of direct evidence related to MNP exposure highlight the need for further translational investigations and well-designed clinical trials to validate in humans the promising findings observed in experimental models.

## 14. Conclusions

This review offers an integrated view of the role of MNPs in biological alteration processes, linking them to the protective potential of food-derived bioactive compounds. The focus is on the ability of these molecules to support the integrity of key biological barriers, modulate the balance of the microbiota, and contribute to maintaining communication between the immune and nervous systems along the oral–gut–brain axis. The functional nutrients analyzed, including icariin, pterostilbene, spirulina, *Opuntia ficus-indica*, artichoke, and quinic acid, exhibit healthy effects in counteracting the oxidative stress and inflammatory processes induced by MNPs, primarily by activating the Nrf2 pathway. This molecular mechanism is a key regulator of cellular resilience responses to environmental stress in maintaining homeostasis. These data support the use of targeted nutritional strategies as a complementary tool to reduce the impact of MNPs and preserve the functionality of the oral–gut–brain axis, opening new perspectives in the field of precision nutrition. Future research should shift from acute, high-dose models to chronic, low-concentration exposure models, which are more representative of real-world conditions. It is necessary to further investigate the accumulation, distribution, and persistence of MNPs along the oral–gut–brain axis. A significant challenge is the limited bioavailability and rapid metabolism of compounds such as icariin and pterostilbene, which may reduce their translational potential despite promising experimental results. Moreover, the lack of standardized dosages and the risk of interactions with drug therapies make careful dose optimization essential. Long-term clinical studies are needed to clarify the alterations in biological barriers and microbiota imbalances associated with prolonged exposure to improve delivery strategies for functional compounds derived from spirulina, *Opuntia ficus-indica*, artichoke, and quinic acid. The use of innovative experimental models, such as three-dimensional platforms, will be crucial in clarifying the complex relationships between environmental factors and human physiology. These developments could pave the way for highly targeted nutritional interventions aimed at modulating the Nrf2 pathway and cellular resilience response, with the ultimate goal of maintaining a functional balance of the oral–gut–brain axis. Furthermore, the use of AI can facilitate the integrated analysis of complex data, contributing to the identification of clinical biomarkers and understanding the interactions between nutrients, microbiota, and cellular resilience pathways. In this new light, AI can support the development of precision nutritional approaches, optimizing the effectiveness of interventions based on bioactive compounds.

## Figures and Tables

**Table 1:** Summary of nutritional modulation of key molecular mechanisms associated with MNP-induced damage in oral–gut–brain axis alterations.

Functional Foods	Molecular Target Inhibited (↓)	Molecular Target Activated (↑)	References
*Artichoke*	NF-κB p65 COX-2 and PGE2 TNF-α, IL-6 and IL-8 STAT3/NF-κB MDA and 8-OHdG NLRP3/caspasi-1/GSDMD	Nrf2 SOD2 CAT GPX4	[114,115,116,117,118]
*Quinic acid*	MDA p38 MAPK	SOD, CAT, GPx Tryptophan Indole-3-acetic acid Kynurenic acid SKN-1/Nrf2 Aβ deposition	[120,121,122,123,124,125,126]
*Opuntia ficus-indica*	ROS TNF-α IL-1β NF-κB	Nrf2 SOD CAT GPX	[127,128,129]
*Spirulina*	TNF-α, IL-6, IL-1β NF-κB MDA	IL-10 Nrf2 PI3K/Akt CAT, SOD and GPX	[130]
*Pterostilbene*	NF-κB iNOS and COX-2 NF-κB/MAPK and TLR4 TNF-α, IL-6, IL-1β	NRF2 HO-1 and NQO1 IL-10 BDNF	[131,132,133,134,135,136,137,138,139]
*Icariin*	IL-1β EphA2-RhoA TLR-4/NF-κB IL-1β, TNF-α, IL-2, IL-6, IL-12	p-Akt and Nrf2 Sirt 1, Pot1α, BUB1b, FOXO1, Ep300, ANXA3, Calb1, SNAP25 BDNF IL-10	[140,141,142,143]

**Table 2:** Summary of probiotic strains and their biological/molecular modulation mechanisms on oral–gut–brain axis alterations.

Probiotic Strain(s)	Biological Context/Model	Biological/Molecular Targets Inhibited (↓)	Biological/Molecular Targets Activated (↑)	Biological Effects Associated	References
*Lactobacillus rhamnosus* SD11	RCT-healthy volunteers	Salivary mutans Streptococci, total salivary bacterial counts	Salivary Lactobacillus levels	Improvement of oral microbiota homeostasis; reduction in cariogenic bacterial burden; promotion of a promotion of a healthier oral environment	[51]
*Lactobacillus* *fermentum* E-3/E-18	In vitro-intestinal isolate from healthy child microbiota	ROS induced oxidative damage, lipid peroxidation	Glutathione content, Mn-SOD, expression, H_2_O_2_ secretion	Enhanced antioxidant defense; antimicrobial activity contributing to intestinal microbial homeostasis	[203]
*Lactobacillus casei* BL23 (CAT/SOD-producing strains)	Murine Chron’s disease model	Oxidative stress, intestinal inflammatory damage	CAT, SOD	Protection of intestinal barrier and attenuation of colitis-associated damage	[204]
*Bacillus subtilis* derived surfactin	Human gingival fibroblasts	Oxidative imbalance	Nrf_2_, HO-1	Activation of periodontal cellular resilience and antioxidants pathways	[205]
*Bifidobacterium animalis* ZK-77 and ZK-88	Rat model of *Porphyromonas* *gingivalis*-induced periodontitis	Growth of oral pathogens biofilm formation H_2_S production NH_3_ production indole production	H_2_O_2_ production, antimicrobial activity	Antimicrobial activity, reduction in cariogenic/ halitosis-associated metabolites, restoration of oral microbiota homeostasis, and attenuation of periodontal inflammation	[206]
*Lactiplantibacillus plantarum* CGMCC 1.16089	AD mouse model	IL-1β, Il-6, TNF-α, ROS, Aβ deposition	Claudin-1, ZO-1	Improved intestinal barrier integrity, attenuation of oxidative stress reduction in neuroinflammation, mitigation of AD pathology	[212]

## Data Availability

No new data were created or analyzed in this study. Data sharing is not applicable to this article.

## Abbreviations

AD	Alzheimer’s disease
Aβ	Amyloid beta peptide
Aβ1–42	Amyloid beta 1–42 peptide
Aβ40/42	Amyloid beta 40/42 isoforms
ABIOME	A Bioreactor Imitation of the Microbiota Environment
AChE	Acetylcholinesterase
ACFB	Artichoke, caihua, and fenugreek vegetal extract original blend
Ac-Tau	Acetylated tau protein
Adcyap1	Adenylate cyclase-activating polypeptide 1
AI	Artificial intelligence
AKT	Protein kinase B (PKB)
pAKT	Phosphorylated AKT
AKT1	Protein kinase B alpha (PKBα)
ALPL	Alkaline phosphatase
AMPK	5′ adenosine monophosphate-activated protein kinase
ANXA3	Annexin A3
AMPs	Antimicrobial peptides
APP	Amyloid precursor protein
APP/PS1	Amyloid precursor protein/presenilin-1 transgenic mouse model
APP/PS2	Amyloid precursor protein/presenilin-2 transgenic mouse model
AREs	Antioxidant-responsive elements
Arg1	Arginasi 1
ASC	Apoptosis-associated speck-like protein containing a CARD
ATG 5/7	Autophagy-related 5/7
BAX	Bcl2-associated X-protein
BALB/c	Bagg albino/c
Bcl2	B-cell lymphoma 2
BBB	Blood–brain barrier
BDNF	Brain-derived neurotrophic factor
BBM	Blood-based biomarker
BMECs	Brain microvascular endothelial cells
BUB1b	BUB1 mitotic checkpoint serine/threonine kinase B
BV2	Immortalized murine microglial cell line derived from C57BL/6 mouse microglia
Calb1	Calbindina 1
Camk2g	Calcium/calmodulin-dependent protein kinase II gamma
CAT	Catalase
Cct4	Chaperonin containing TCP1 subunit 4
CD	Crohn’s disease
CD86	Cluster of Differentiation 86
CFP	Complement factor properdin
cGMP	Cyclic guanosine monophosphate
CNS	Central nervous system
COX-2	Cyclooxygenase-2
CSF	Cerebrospinal fluid
CTSK	Catepsina K
CREB	cAMP response element-binding protein
DSS	Dextran sodium sulfate
Endog	Endonuclease G
EP300	E1A binding protein p300
EphA2	Ephrin type-A receptor 2
EVs	Extracellular vesicles
FABP3	Fatty acid-binding protein 3
FOS	Fructooligosaccharides
FOXO	Forkhead box O
FOXO1	Forkhead box protein O1
GABA	Acido gamma-amminobutirrico
GAPDH	Gliceraldeide-3-fosfato deidrogenasi
γ-GCs	γ-glutamilcisteina sintetasi
GFs	Gingival fibroblasts
GLUT2	Glucose transporter 2
GOS	Galactooligosaccharides
GOT1	Glutamate oxaloacetate transaminase 1
GPX	Glutathione peroxidase
GPX4	Glutathione peroxidase 4
GSDMD	Gasdermin D
GSH	Glutathione
GSR	Glutathione reductase
Hbq1a	Hemoglobin, theta 1A
H2O2	Hydrogen peroxide
HMGB1	High-mobility group box 1
HO-1	Heme oxygenase-1
HPG	Highly porous gold electrodes
hs-CRP	High-sensitivity C-reactive protein
H_2_S	Hydrogen sulfide
Hsp70	Heat Shock Protein 70
5-HT	5-hydroxytryptamine
IAA	Indole-3-acetic acid
IBD	Inflammatory bowel disease
ICA	Icariin
ICT	Icaritin
ICS	Icariside II
IEB	Intestinal epithelial barrier
IEC	Intestinal epithelial cells
IgA	Immunoglobulin A
iNOS	Inducible nitric oxide synthase
iNPH	Idiopathic normal pressure hydrocephalus
IL	Interleukin
IL-1β	Interleukin-1 beta
IL-4	Interleukin-4
IL-6	Interleukin-6
IL-8	Interleukin-8
IL-10	Interleukin-10
IL-12	Interleukin-12
IL-18	Interleukin-18
JNK	c-Jun N-terminal kinase
JPH3	Junctophilin-3
KDR	Kinase insert domain receptor
Keap1	Kelch-like ECH-associated protein 1
LASSO	Least Absolute Shrinkage Operator
LC3	Microtubule-associated proteins 1A/1B light chain 3
LDH	Layered double hydroxide
LPS	Lipopolysaccharide
MAP9 (Mtap9)	Microtubule-associated protein 9
MAPK	Mitogen-activated protein kinase
MAPK14	Mitogen-activated protein kinase 14
MAPT	Microtubule-associated protein tau
MARS	Multivariate adaptive regression splines
MCI	Mild cognitive impairment
MDA	Malondialdehyde
MLKL	Mixed lineage kinase domain-like pseudokinase
MMP9	Matrix metalloproteinase-9
MNPs	Micro- and nanoplastics
MPs	Microplastics
mRNAs	Messenger RNAs
mTOR	Mechanistic target of rapamycin
mTORC1	Mammalian/mechanistic target of rapamycin complex 1
Muc2	Mucin 2
NAAA	N-acylethanolamine acid amide hydrolase
NAD^+^	Nicotinamide adenine dinucleotide
NAT2	N-acetyltransferase 2
NCM460	Human normal colon mucosal epithelial cell line
NF-κB	Nuclear factor kappa B
Nfe2l2	Nuclear factor erythroid 2-related factor 2 gene
NfL	Neurofilament light chain
NH_3_	Ammonia
NLRP3	NOD-, LRR-, and pyrin domain-containing protein 3 inflammasome
NMN	Nicotinamide mononucleotide
NO	Nitric oxide
NPs	Nanoplastics
NQO1	NAD(P)H quinone oxidoreductase 1
Nrf2	Nuclear factor erythroid 2-related factor 2
OEB	Oral epithelial barrier
OFI	*Opuntia ficus-indica*
8- OHdG	8-Hydroxy-2′-deoxyguanosine
p53	Tumor protein p53
p62	p62 protein or sequestosome-1
p65	Nuclear factor NF-kappa-B p65 subunit
p-p65	Phosphorylated NF-κB p65 subunit
PD	Parkinson’s disease
PDE4B	Phosphodiesterase 4B
PE	Polyethylene
PEG	Polyethylene glycol
PET	Polyethylene terephthalate
Per1	Period circadian regulator 1
PFKFB3	6-phosphofructo-2-kinase/fructose-2,6-bisphosphatase 3
PGC-1α	Peroxisome proliferator-activated receptor gamma coactivator 1-alpha
PGE2	Prostaglandin E2
PHA	Phytohemagglutinin
PIM2	Proviral integration site for Moloney murine leukemia virus 2
PI3K	Phosphoinositide 3-kinase
Pik3cg	Phosphoinositide-3-kinase catalytic gamma polypeptide
PLA2G1B	Phospholipase A2, group 1B
p-mTOR	Phosphorylated mechanistic target of rapamycin
Pot1α	Protection of telomeres 1 alpha
PP	Polypropylene
PPARγ	Peroxisome proliferator-activated receptor gamma
PRKCG	Protein kinase C gamma type
Prnp	Prion protein gene
PS	Polystyrene
PSA	Plasma scavenging activity
PS-COOH	Carboxyl-modified polystyrene microplastics
p-tau181	Phosphorylated tau protein 181
p-tau217	Phosphorylated tau protein at threonine 217 residue
PTB	Pterostilbene
PTFS2	Prostaglandin-endoperoxide synthase 2
p-ULK1	Phosphorylated Unc-51-like autophagy-activating kinase 1
PVC	Polyvinyl chloride
QA	Quinic acid
RAGE	Receptor for advanced glycation end products
RANKL	Receptor activator of nuclear factor κB ligand
RhoA	Ras homolog family member A
ROS	Reactive oxygen species
RTP4	Receptor transporter protein 4
SCFAs	Short-chain fatty acids
SCG3	Secretogranin-3
SGLT1	Sodium-glucose cotransporter 1
SH-G	Sulfhydryl groups
SIRT1	Sirtuin 1
SIRT6	Sirtuin 6
SKN-1	Skinhead-1 transcription factor
Slc7a5	Solute carrier family 7 member 5
SOD	Superoxide dismutase
SOD1	Superoxide dismutase 1 (Cu/Zn superoxide dismutase)
SOD2	Superoxide dismutase 2 or manganese superoxide dismutase
SNAC	Alpha-synuclein
SNAP25	Synaptosome-associated protein 25
SP	Spirulina (Arthrospira platensis)
SPP1	Secreted phosphoprotein 1
STAT3	Signal transducer and activator of transcription 3
SULT1A1	Sulfotransferase 1A1
Th1	T helper 1
Th2	T helper 2
Th17	T helper 17
TJs	Tight junction proteins
TLR-2	Toll-like receptor 2
TLR4	Toll-like receptor 4
TNF-α	Tumor necrosis factor alpha
TPI1	Triosephosphate isomerase 1
Tregs	Regulatory T cells
Trx	Thioredoxin
UC	Ulcerative colitis
UGT2B4	UDP-glucuronosyltransferase family 2 member B4
ULK1	Unc-51-like autophagy-activating kinase 1
UVA	Ultraviolet A
Vegfd (Figf)	Vascular endothelial growth factor D
VGF	Nerve growth factor-inducible
Zfand5	Zinc finger AN1-type containing 5
ZO-1	Zonula occludens-1
